# Genome Analysis Identified Novel Candidate Genes for Ascochyta Blight Resistance in Chickpea Using Whole Genome Re-sequencing Data

**DOI:** 10.3389/fpls.2017.00359

**Published:** 2017-03-17

**Authors:** Yongle Li, Pradeep Ruperao, Jacqueline Batley, David Edwards, Jenny Davidson, Kristy Hobson, Tim Sutton

**Affiliations:** ^1^School of Agriculture, Food and Wine, University of Adelaide, AdelaideSA, Australia; ^2^School of Agriculture and Food Sciences, University of Queensland, BrisbaneQLD, Australia; ^3^School of Plant Biology and Institute of Agriculture, University of Western Australia, CrawleyWA, Australia; ^4^South Australian Research and Development Institute, UrrbraeSA, Australia; ^5^New South Wales Department of Primary Industries, TamworthNSW, Australia

**Keywords:** association study, re-sequencing, ascochyta blight, disease resistance, selective sweep, Fst, QTL

## Abstract

Ascochyta blight (AB) is a fungal disease that can significantly reduce chickpea production in Australia and other regions of the world. In this study, 69 chickpea genotypes were sequenced using whole genome re-sequencing (WGRS) methods. They included 48 Australian varieties differing in their resistance ranking to AB, 16 advanced breeding lines from the Australian chickpea breeding program, four landraces, and one accession representing the wild chickpea species *Cicer reticulatum*. More than 800,000 single nucleotide polymorphisms (SNPs) were identified. Population structure analysis revealed relatively narrow genetic diversity amongst recently released Australian varieties and two groups of varieties separated by the level of AB resistance. Several regions of the chickpea genome were under positive selection based on Tajima’s *D* test. Both Fst genome- scan and genome-wide association studies (GWAS) identified a 100 kb region (AB4.1) on chromosome 4 that was significantly associated with AB resistance. The AB4.1 region co-located to a large QTL interval of 7 Mb∼30 Mb identified previously in three different mapping populations which were genotyped at relatively low density with SSR or SNP markers. The AB4.1 region was validated by GWAS in an additional collection of 132 advanced breeding lines from the Australian chickpea breeding program, genotyped with approximately 144,000 SNPs. The reduced level of nucleotide diversity and long extent of linkage disequilibrium also suggested the AB4.1 region may have gone through selective sweeps probably caused by selection of the AB resistance trait in breeding. In total, 12 predicted genes were located in the AB4.1 QTL region, including those annotated as: NBS-LRR receptor-like kinase, wall-associated kinase, zinc finger protein, and serine/threonine protein kinases. One significant SNP located in the conserved catalytic domain of a NBS-LRR receptor-like kinase led to amino acid substitution. Transcriptional analysis using qPCR showed that some predicted genes were significantly induced in resistant lines after inoculation compared to non-inoculated plants. This study demonstrates the power of combining WGRS data with relatively simple traits to rapidly develop “functional makers” for marker-assisted selection and genomic selection.

## Introduction

Chickpea (*Cicer arietinum*) is one of the world’s most important grain legumes providing protein and micronutrients for millions of people in developing countries. Chickpea is an important commodity crop in Australia with a total production of 0.7 million ton in 2012 (FAO, 2012) and is an essential rotation component of farming systems providing nutritional benefits through nitrogen fixation and disease break. There are two market types of chickpea: kabuli and desi which difference in seed color, seed shape, and flower color. Following India, Australia is the world’s second largest producer of chickpea; much of the annual harvest is exported to the Indian sub-continent.

Ascochyta blight (AB) is caused by the fungal pathogen *Ascochyta rabiei* (Pass.) Labr. AB symptoms can occur in any parts of the plant above the ground at any growth stage depending on the availability of the pathogen and the right environmental conditions. Infection can lead to necrotic lesions on leaves, stem breakage and eventual death of the plant as well as pod abortion and seed staining (Pande et al., 2005). The chickpea growing area in Australia reduced from 260,000 ha in 1998 to 110,000 ha in 2006 largely due to lack of durable AB resistance in commercial varieties and loss of growers’ confident in growing chickpea (FAO, 2012). A similar decline in chickpea production caused by the outbreak of AB has also occurred in Canada (Chandirasekaran et al., 2009), USA (Kaiser et al., 1994), and Latin America (Kaiser et al., 2000). *A. rabiei* is spread by wind and rain splash, can survive on infected stem for up to 20 months (Kaiser and Hannan, 1987) and in artificial conditions has also been shown to be pathogenic on cowpea, lentil, and field pea (Pande et al., 2005). AB can be effectively controlled via intensive fungicide application, implementation of crop rotation strategies and seed treatment; however, using varieties with improved resistance remains one of the most cost-effective ways to manage AB in chickpea. The first Australian cultivar with improved resistance to AB compared to current varieties at the time, was Howzat released in 2001, followed by, Flipper, Yorker, and the most significant improvement with Genesis090 in 2005. As a result of selective breeding for AB resistance in chickpea, current varieties that make up the majority of annual chickpea production in Australia are rated as moderately resistant or resistant although loss of resistance was observed in a number of cultivars in 2016 (SA Sowing Guide 2017).

Using conventional breeding methods, considerable progress has been made towards the improvement of AB resistance in chickpea varieties (Pande et al., 2005). The application of marker-assisted breeding has recently gained in momentum due to the fast declining cost of genotyping/sequencing technologies and the emergence of high-throughput automatic technology. Using traditional bi-parental populations, several QTL for AB resistance have been identified on linkage groups LG2 (Udupa and Baum, 2003; Cho et al., 2004), LG3 (Tar’an et al., 2007), LG4 (Lichtenzveig et al., 2006; Tar’an et al., 2007; Sabbavarapu et al., 2013; Stephens et al., 2014), LG5 (Sabbavarapu et al., 2013), LG6 (Tar’an et al., 2007; Sabbavarapu et al., 2013), and LG8 (Lichtenzveig et al., 2006). One major QTL and/or one minor QTL have been repeatedly reported in a similar region of LG4 across several studies and therefore make this locus a good candidate region for improving AB resistance in chickpea (Lichtenzveig et al., 2006; Tar’an et al., 2007; Sabbavarapu et al., 2013; Stephens et al., 2014). Madrid et al., have identified an AB resistance gene, ethylene receptor (*ETR-1*), located near the peak of the major QTL in LG4 flanked by markers NCPGR91 and GAA47 (Madrid et al., 2013). Transcriptional profiling using 756 microarray features identified 95 candidate genes differentially expressed during *A. rabiei* infection in four chickpea genotypes (Coram and Pang, 2006). However, the *ETR-1* candidate characterized by Madrid et al. (2013) was not identified as being differentially expressed in the study by Coram and Pang (2006). A recent published study by Leo et al. (2016) examined the expression profiles of seventeen candidate genes. This work showed that six genes were differentially expressed across ten host genotypes under AB infection; however, their expression levels did not correlate well with the resistance classification of the lines suggesting that they might have a minor role in AB resistance and hence further research is warranted.

Next-generation sequencing technology can provide a relatively cheap and high-throughput genotyping option to discover genome variation and identify selection signatures in crop species such as chickpea (Varshney et al., 2013). Genotyping using molecular markers has been one of the cornerstone developments in modern plant breeding. There are now many methods that utilize NGS for genotyping, such as reduced genome representation sequencing methods like RAD-seq, GBS, and whole-genome resequencing (WGRS) (Davey et al., 2011; Elshire et al., 2011). These methods have different advantages and disadvantages and thus are suitable for different applications. Compared to WGRS, RAD-seq, and GBS are cheaper as they sample only a fraction of the genome. Thus, these methods are suitable for large scale genotyping of crops with large genome sizes, for example, genotyping a large number of F2 or advanced lines in a breeding program. In contrast, WGRS is more suited to pre-breeding activities where smaller number of key elite parents, landraces and wild species need to be studied carefully for genome variation (SNPs, CNV, structural variation) and association studies (Li et al., 2011). The cost of sequencing has dropped rapidly in the last decade, however, the major cost and time consuming part of NGS remaining is library preparation (Rohland and Reich, 2012). Fortunately, automation of library preparation using liquid-handling robotic stations is developing rapidly and now available from several commercial companies. Many WGRS studies have been reported in crop species such as rice (Huang et al., 2012; Wang et al., 2014), sorghum (Mace et al., 2013), tomato (Lin et al., 2014), and chickpea (Lake et al., 2016; Sadras et al., 2016; Thudi et al., 2016). One of the common findings in these studies is the marked reduction of genomic variation during domestication and/or breeder’s selection.

The first objective of this study was to investigate the effect of selective breeding (AB resistance) on genetic diversity and population structure of the Australian chickpea breeding program over the last four decades using WGRS approaches. Secondly was to identify candidate genes involved in AB resistance associated with a major QTL on chromosome 4 using Fst genome-scan and genome-wide association mapping approaches. Finally, results were validated using an independant set of chickpea germplasm and qPCR analysis.

## Materials and Methods

### Plant Materials and Sequencing

In this study, the plant materials include 48 chickpea varieties released in Australia from 1978 to 2016, 16 advanced breeding lines, four landraces, and one wild chickpea *C. reticulatum* (Supplementary Table S1). The released varieties and advanced breeding lines are a good representation of the genetic diversity present in the Australian chickpea breeding program. The wild species *C. reticulatum* and landraces serve as a reference point for investigating genetic diversity. DNA was extracted from young leaf using Qiagen DNeasy Plant Mini Kit according to the manufacturer’s instructions. Pair-end sequencing libraries were constructed for each genotype with insert sizes of ∼500 bp using TruSeq library kit according to the Illumina manufacturer’s instruction. Around 40 million 150 bp paired-end reads for each genotype were generated by the Australian Genome Research Facility in Brisbane, Australia using Illumina HiSeq 2000 platform. Sequence data is available from the NCBI Short Read Archive under BioProject accession PRJNA375953.

### Population Genomics Analysis

Paired-end reads for each genotype were trimmed, filtered, and mapped to the kabuli reference genome 1.0 using SOAP2 (Li et al., 2009). SNPs were called using the SGSautoSNP pipeline (Lorenc et al., 2012). The BAM files of each cultivar were separated into 16 AB resistant and 24 susceptible genotypes as two contrasting groups to obtain sample allele frequencies (SAF file) which is the probability of all read data given the sample allele frequency using the software ANGSD (Korneliussen et al., 2014). The resulting two SAF files of the two contrasting groups were used to estimate joint distribution of sample allele frequencies (2D-SFS) which was used as prior together with the two SAF files in Fst estimation using software ngsPopGen (Fumagalli et al., 2013). To reduce the effect of sampling error, Fst values of each site (SNPs) within a 100 kb non-overlapping window were averaged. The whole genome was scanned to identify regions with extreme population genetic differentiation (large Fst value compared to the surrounding region) which could be served as an indicator of selection signature. The rationale is that genetic differentiation between groups at a given neutral locus is determined by stochastic random factors such as genetic drift. If a locus is under natural or artificial selection, the pattern of genetic differentiation may change. For example, regions showing uncommon large amounts of genetic differentiation (difference alleles are fixed in different groups) may have undergone diversifying selection.

To correct errors in NGS data, allele frequencies were estimated using site frequency spectrum (SFS) as prior to improve inference of population genetic parameters (𝜃π, 𝜃w, and Tajima’s *D*) using the software ANGSD (Korneliussen et al., 2014). Nucleotide diversity (𝜃π) was calculated separately for 16 AB resistant and 24 susceptible genotypes. The resistant genotypes were released after 2005 while the susceptible genotypes were released before 2005 except for GenesisKalkee and PBAPistol. To investigate directional and balancing selection in the chickpea genome, the SFS based neutrality test Tajima’s *D* was calculated in 100 kb non-overlapping windows using the 69 genotypes (Korneliussen et al., 2013).

The relationship of the 69 genotypes was visualized using principal components analysis (PCA) implemented in ngsPopGen, a modification of Patterson’s approach of PCA where SFS was incorporated to reduce uncertainty of genotype calling (Fumagalli et al., 2014).

Genome-wide association studies was performed using 59 genotypes with AB resistance data obtained from the Australian chickpea breeding program from evaluation over multiple years and locations. Mixed linear models (MLM) that implemented in the software GAPIT was used to evaluate the effects of each ∼250,000 SNPs (MAF > 5%) individually, adjusting for confounding effect such as population structure and kinship (Lipka et al., 2012). In order to speed up the computation time, the kinship matrix was compressed to its optimum groups and P3D method (population parameters previously determined) was used. The MLM can be written as:

(1) $$\begin{array}{c} y\;=\;1\beta_{1}+X_{SNP}\beta_{SNP}+Q_{PCA}V_{PCA}+\\ \;Z_{GENOTYPE}\gamma_{GENOTYPE}+\varepsilon ,\;\left(1\right) \end{array}$$

where *y* is the *n* × 1 vector of AB scores, *1* denotes a *n* × 1 vector of 1s and *β_*1*_* is the intercept, ***X_SNP_*** (*n* ×*p*) is design matrix for the fixed effects of SNPs, ***Z_GENOTY PE_*** (*n* ×*h*) is the corresponding design matrix for the random effects of genotype, ***Q_PCA_*** is design matrix for the fixed effects of population structure. The random genotype effect was similarly assumed to follow a normal distribution, *γ_GENOTY PE_* ∼ N (0, ***K***σ^2^_g_), where ***K*** was the estimated kinship matrix and σ^2^_g_ the variance component due to genotype. To account for kinship in the estimation of random genotype effects, γ*_GENOTY PE_*, the design matrix ***Z_GENOTY PE_*** was multiplied by the cholesky-root of the kinship matrix. The residual error vector *𝜀* (*n* × 1) was assumed to comprise independent and identically distributed random normal errors with mean of 0 and variance σ^2^, *𝜀*∼ N (0, ***I***σ^2^).

The significant *p*-value cut-off was set as 3. 47E-04. Setting a *p*-value cut-off as 2.00E-07 (0.05/250,000) using the Bonferroni correction is too conservative for a pilot study with a relative small sample size like the current study. Besides, Bonferroni correction assumes the test variables are independent whereas SNPs are not independent due to Linkage disequilibrium (LD). Therefore, a modified Bonferroni correction was used in this study; an alpha level of 0.05 is divided by the number of independent segments of the genome (instead of the number of tested SNPs) which is calculated from the average decay of LD in this germplasm. The average decay of LD in this study (*r*^2^= 0.2) is 5,062 kb, given the chickpea genome size of 730,000 kb, the number of independent segments of the genome in this germplasm is 144. Therefore, the p-value cut-off was set as 0.05/144 which is 3. 47E-04. The circular representation of the chickpea genome was generated using software CIRCOS (Krzywinski et al., 2009)

### GWAS Validation

A panel of 132 advanced chickpea lines from diverse backgrounds was used for validation. In order to evaluate AB resistance, the 132 advanced lines were grown with RCBD design and replicated two times in pots with four plants in an open area enclosed by a net to avoid animal damage. The seedlings were inoculated to run off with a single conidium-derived *A. rabiei* isolate (FT 13092-1, at a concentration of 1 × 10^6^ spores/ml) when plants were 5 weeks old. This isolate which belongs to pathotype IV was collected in 2013 from Genesis 090 chickpea (one of the tested lines) in a trial at Kingsford Research Station, South Australia. Plants were kept with an optimal moisture level by mist irrigation. Three weeks after inoculation, AB resistance scores were measured by carefully examining the level of damage on leaves and stems of each plant using with a disease rating scale of 1–9 modified from Singh et al. (1981). The 132 advanced chickpea lines were sequenced and SNPs were called in the same way as the 69 genotypes described above. GWAS was also performed in the same way as the 59 genotypes using GAPIT.

### Quantitative Real-Time PCR (qPCR)

Quantitative Real-Time PCR (qPCR) was performed on six chickpea lines of differing AB resistance (PBAPistol, DICC8191, PBAMonarch, ICC3996, ICC12004, DICC8218) from the panel of 132 advanced lines under the condition of with and without (mock-treated) *A. rabiei* inoculation. Leaf tissues of the six genotypes (5 weeks old stage) were collected 24 and 48 h after inoculation with six biological replicates taken. RNA was isolated and purified using Direct-zol RNA Miniprep according to the manufacturer’s instructions. cDNA synthesis was carried out using SuperScript^®^ IV Reverse Transcriptase (Life Technologies). The cDNA samples were diluted 20 times in MQ H2O. Three replicate PCRs for each of the samples were included in every run containing: 2 μL of cDNA solution (or the diluted standard, or water), 5 μL Kapa Sybr Fast Universal 2X qPCR Master Mix (Geneworks), 1.2 μL of each of the forward and reverse primers (Supplementary Table S2) at 4 μM and 0.6 μL of water. The total volume of the PCR reactions was 10 μL. Reactions were performed in QuantStudio6 (Life Technologies): 3 min at 95°C followed by 40 cycles of 3 second at 95°C, 20 s at 60°C, fluorescent acquisition at 60°C. Followed by melt curve analysis: 15 s at 95°C, 1 min at 60°C then increase temperature from 60°C to 95°C with fluorescence readings acquired at 0.5°C increments. Three reference genes (HSP90, EF1a, GAPDH), determined to be expressed consistently previously, were used to normalize the expression level of candidate genes (Garg et al., 2010).

## Results

### Genome Variation

Sixty-nine chickpea genotypes were sequenced using WGRS methods. They included 47 chickpea varieties released in Australia from 1978 to 2013, 17 advanced breeding lines, four landraces and one accession representing the wild chickpea species *Cicer reticulatum* (Supplementary Table S1). In total, approximately 0.9 billion Illumina paired-end reads (150 bp) from 69 genotypes were mapped to the kabuli reference genome 2.6.2. The mapping depth ranged from 0.64× to 10.37× with a mean of 3.35×. For the 69 genotypes, 827,411 SNPs ranging from 170,747 on Ca4 to 28,624 on Ca8 were discovered (**Table 1**). However, when the *C. reticulatum* accession PI48977 was removed from analysis the total number of SNPs dropped to 444,359, while 𝜃π dropped from 1.07 × 10^-4^ to 0.83 × 10^-4^ (**Table 2**). Further, excluding the four landraces from the analysis (leaving 64 varieties and advanced breeding lines), the total number of SNPs only dropped from 451,546 to 429,810, while 𝜃π dropped from 0.83 × 10^-4^ to 0.81 × 10^-4^, which indicated that the varieties and advanced breeding lines represented most of the genetic diversity present in the landraces included in this study. When the collection of 47 varieties was grouped into release dates from 1978-2004 (predominantly AB susceptible) and 2005–2013 (predominantly AB resistant), it was shown that the latter represented a lower level of genetic diversity (**Table 2**). LD was estimated using 17,905 high-confident SNPs with minimum coverage of five reads. The r^2^ on each chromosome ranged from 0.07 to 0.28 with an average of 0.14 (**Table 1**). Setting *r*^2^ cut-off as 0.2, LD decay ranged from 500 to 23,000 kb with an average of 5,062 kb (**Table 1** and Supplementary Figures S1A–H).

**Table 1:** Summary of linkage disequilibrium (LD) and single nucleotide polymorphisms (SNPs) used to estimate LD.

Chromosome	No. SNPs in 69 genotypes^1^	No. SNPs in 68 genotypes^2^	No. SNP used to estimated LD	Mean *r*^2^	LD decay (kb)
Ca1	110,295	69,424	3,386	0.18	2,000
Ca2	75,410	40,404	2,667	0.10	1,500
Ca3	105,954	42,213	1,444	0.15	4,800
Ca4	170,747	118,778	4,092	0.28	23,000
Ca5	112,194	46,770	1,457	0.07	1,000
Ca6	130,732	68,481	2,276	0.10	2,500
Ca7	93,455	44,933	2,316	0.16	5,200
Ca8	28,624	13,356	267	0.08	500
Total/average	827,411	444,359	17,905	0.14	5,062

*^1^68 *Cicer arietinum* plus one Cicer reticulatum*

*^2^68 Cicer arietinum*

**Table 2:** Genetic diversity of the 69 genotypes.

Germplasm	No. genotypes	No. SNPs	𝜃_π_(10^-4^)
Varieties+advanced Lines+landraces+wild	69	827,411	1.07
Varieties+advanced Lines+landraces	68	451,546	0.83
Varieties+advanced lines	64	429,810	0.81
Varieties	47	312,955	0.73
–Released during 1978–2004 (AB susceptible predominantly)	21	233,059	0.78
–Released during 2005–2013 (AB resistant predominantly)	16	162,748	0.59

### Population Structure

**FIGURE 1:**
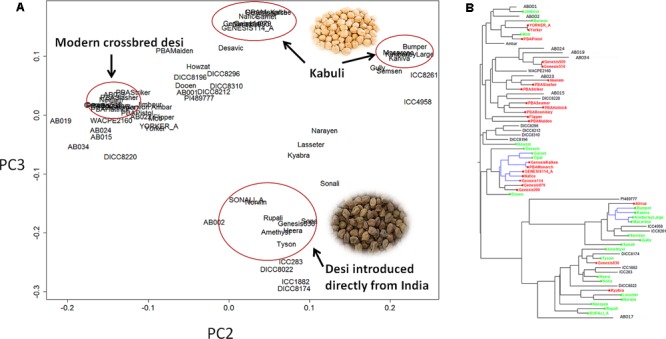
**Population structure of 69 chickpea genotypes based on single nucleotide polymorphism (SNP) data. (A)** Principle component analysis (PCA). **(B)** Phylogenetic tree. Varieties released after 2005 [mainly ascochyta blight (AB) resistant] are highlighted in red, varieties released before 2005 (mainly AB susceptible) are highlighted in green, the remainder are advanced lines or landraces. The tree branches highlighted in blue are kabuli, whereas the rest are desi.

Principle component analysis (PCA) showed that the *C. reticulatum* accession PI48977 separated clearly from *C. arietinum* under PC1 vs. PC2 (Supplementary Figure S2). In PC2 vs. PC3, there were two distinct groups of kabuli chickpea whereas the relationship of desi chickpea was more complex (**Figure 1**). One group of kabuli mainly contained the Genesis^TM^ series introduced to Australia from ICARDA (International Center for Agricultural Research in the Dry Areas). The other grouping of kabuli mainly contained older released varieties dating back to the 1980’s, with unknown origin. The desi types were generally separated from the kabuli types with a few exceptions (Gully, Semsen). One group of desi type contains lines introduced directly from ICRISAT (International Crops Research Institute for the Semi-Arid Tropics) and their progeny. This group includes some old Australian cultivars such as Tyson, Amethyst, Sona, Heera, and Norwin. Rupali and Sonali, derived from Amethyst and Tyson, respectively, also belong to this group and have gone through a pollen selection process at low temperature aimed at developing chilling tolerant varieties. Another group of desi lines, containing modern variety releases from the Australia chickpea breeding program, cluster closely together and have very narrow genetic diversity (**Figure 1** and **Table 2**). In fact, most of the recently released desi varieties (PBAMaiden, PBAStriker, PBABoundary, PBASlasher, PBAHattrick, PBASeamer, Neelam, and Ambar) have their pedigree traced back to ICC3996, ICC14903, and ICC13729; three AB resistant lines from Iran. The Phylogenetic tree was in agreement with the PCA in general. Varieties released prior to and after 2005 were separated into two distinct groups. The significant outbreak of AB in Australia in the late 1990s that led to rapid decline in area sown to chickpea initiated the rapid prioritization of breeding for improved ascochyta resistance (Pande et al., 2005). As such, varieties released after 2005 were predominantly AB resistant and varieties released before 2005 were predominantly AB susceptible.

### Selection Signature and AB Resistance

**FIGURE 2:**
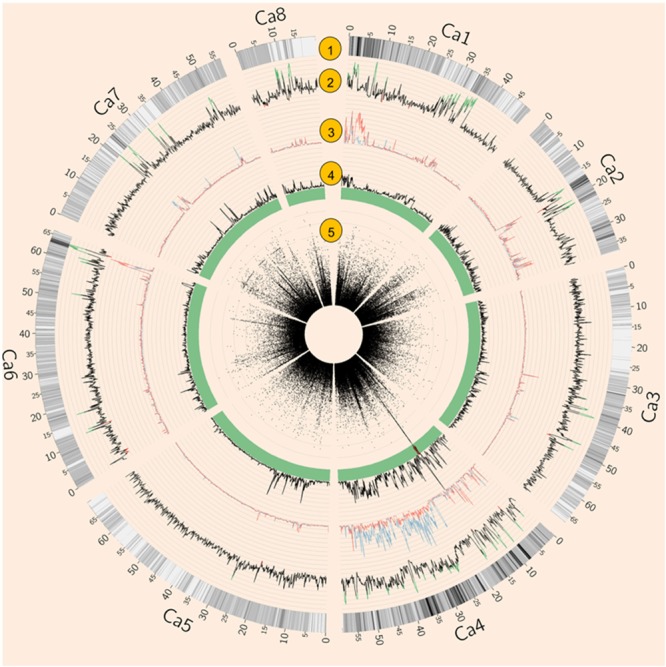
**Summary of whole genome re-sequencing (WGRS) data.** (1) SNP density. (2) Tajima’s *D* of 64 Australian varieties and four Indian landraces. Values above 2 are highlighted in green while values below –2 are in red. (3) Nucleotide diversity (𝜃π) of AB susceptible (blue) and resistant (red) varieties. (4) Fst of AB susceptible versus resistant varieties. (5) Circular Manhattan plot of genome-wide association studies (GWAS) result. Each black dot represents a SNP, Red dots represent SNPs with *p*-values lower than 3. 47E-04 (equal to 0.05 with modified Bonferroni correction).

Both natural and artificial selection shape the chickpea genome, and methods such as Tajima’s D have been widely used to detect selection signatures in genomes (Qanbari and Simianer, 2014). To avoid biased estimation of allele frequency using low depth NGS data, Tajima’s *D* was calculated using an empirical Bayes approach (Korneliussen et al., 2013). Tajima’s *D* showed that 4.74% of the genome was under balancing selection (*D* > 2) while 0.66% of the genome was under purifying selection (*D* < –2, **Figure 2**). Chromosome 1 had the largest proportion (11.22%) of genome under balancing selection whereas chromosome 5 had the least (0.14%). Chromosome 8 had the largest proportion (2.5%) of the genome under purifying selection, whereas none was detected on chromosome 7 (**Figure 2** and Supplementary Table S3).

There were 730 predicted genes under balancing selection (*D* > 2) of which 427 genes have been deposited in the KEGG database^1^ and classified into ten functional categories: genetic information processing (210), environmental information processing (42), carbohydrate metabolism (26), enzyme families (18), amino acid metabolism (16), cellular processes (15), lipid metabolism (15), energy metabolism (12), other categories (49), and unclassified (24). We observed 21 NBS-LRR genes and 98 receptor-like kinases (RLK) under balancing selection, comprising 16.3% of total genes under balancing selection. NBS-LRR and RLK are well known classes of resistance genes in plants and a target of balancing selection (McDowell et al., 1998). However, we did not find any pathway enriched with genes under selection using the web-based software KOBAS (Wu et al., 2006).

There were 171 predicted genes under purifying selection (*D* < –2), of which 90 genes have been deposited in the KEGG database and classified into ten functional categories: genetic information processing (35), environmental information processing (10), carbohydrate metabolism (9), amino acid metabolism (5), cellular processes (9), nucleotide metabolism (3), Metabolism of terpenoids and polyketide (3), lipid metabolism (3), other categories (8), and unclassified (4). Three genes under purifying selection (beta-amyrin 11-oxidase, gibberellin 2-beta-dioxygenase, transcription factor *PIF3*) are involved in gibberellic acid biosynthesis and signal transduction. Additionally, two genes *AUX/IAA* and *JAZ* were involved in auxin and jasmonic acid signal transduction, respectively. However, we did not find any pathways enriched with genes under selection using the web-based software KOBAS (Wu et al., 2006). In contrast to the large proportion of NBS-LRR and RLK candidate genes observed under balancing selection, a single NBS-LRR gene and four RLK genes were identified under purifying selection.

**FIGURE 3:**
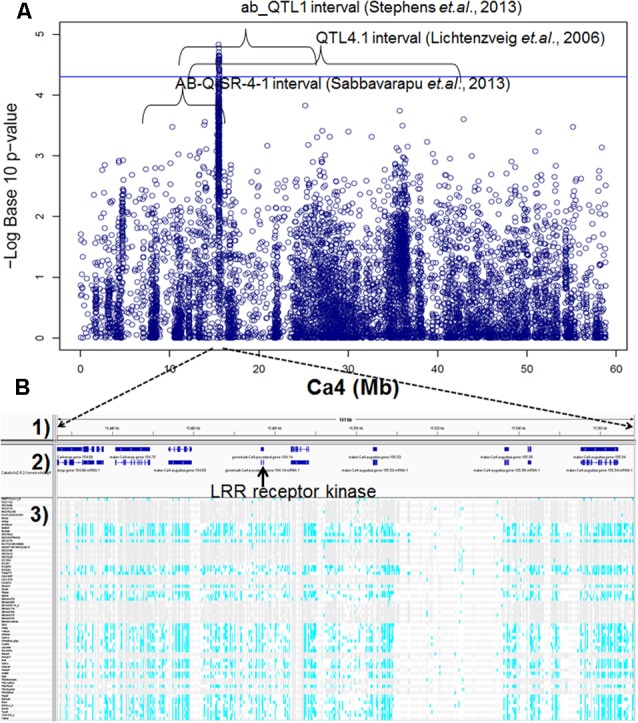
**(A)** Manhattan plot of GWAS result on Ca4. Twenty SNPs, significantly associated with AB resistance, were located within three AB resistant QTL intervals discovered previously by bi-parental mapping. **(B)** Predicted genes and SNPs in AB4.1 associated with AB resistance. (1) Physical position. (2) Predicted genes. (3) SNPs of 69 genotypes in AB4.1, gray represents reference allele, light blue represents alternative allele, and white represents missing.

**FIGURE 4:**
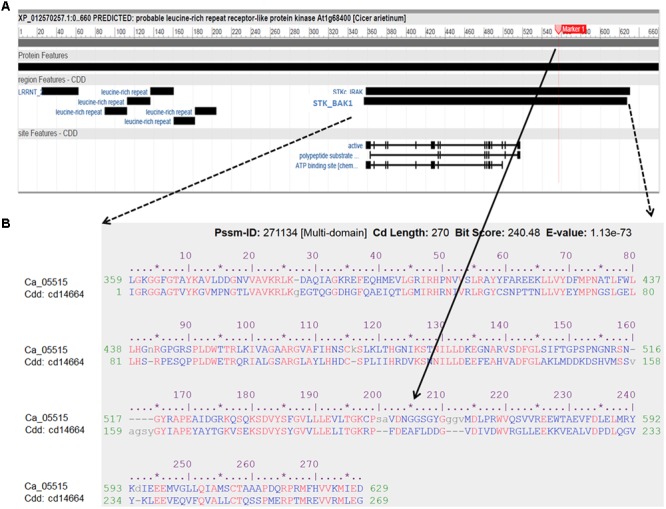
**Characterization of the LRR receptor-like kinase (Ca_05515/XP012570257) and its conserved domains cd14664. (A)** Schematic diagram of the protein features of the LRR receptor-like kinase. Black boxes indicated specific conserved domain regions. LRRNT: N-terminal domain of LRR; STK_BAK1: kinases domain of brassinosteroid (BR)-associated kinase (BAK1). **(B)** Alignment of amino acid sequence of the LRR receptor-like kinase with the best hit conserved domain STK_BAK1. The SNP (Ca4: 15,920,939) with significant association with AB resistance, located in the STK_BAK1 leading to amino acid substitution (Gly/Ala), is highlighted as Marker 1 and indicated in the alignment.

Genome-wide association studies identified 20 SNPs significantly (*p* < 0.001) associated with AB resistance explaining 19.8–21.8% phenotypic variation (**Figure 2** and Supplementary Table S5). These SNPs in high LD were all clustered into a peak on chromosome 4 (Ca4: 15,855, 018..15,980,584), called AB4.1 hereafter. In total, 12 predicted genes were located in the AB4.1 region including one LRR receptor-like kinase (Ca_05515), one wall-associated kinase (Ca_05520), one zinc finger protein (Ca_05511), one cysteine-rich receptor-like kinase (Ca_05516), four serine/threonine protein kinases (Ca_05517, Ca_05521, Ca_05522, and Ca_05523) and five uncharacterized proteins (Ca_05512, Ca_05513, Ca_05514, Ca_05518, and Ca_05519, **Figure 3** and Supplementary Table S4). One significant SNP (Ca4: 15,920,939), located in the conserved catalytic domain of the LRR receptor-like kinase (Ca_05515), led to an amino acid substitution (Gly/Ala, **Figure 4**). All other significant SNPs were located in non-coding regions of the genes. The chickpea genome was scanned to identify selection signatures of AB resistance using the Fst outlier-based approach. A sliding window of 100 kb was used to minimize the effect of sampling error. Fst compares the variance of allele frequencies within and between ABS and ABR groups. The distribution of Fst was highly skewed toward 0, but ranged from 0 to 0.84 across the whole genome (Supplementary Figure S3). Chromosome 4 had the largest average Fst (0.14) while Chromosome 6 had the smallest average Fst (0.03). The region with the largest Fst (0.84) was located on chromosome four spaning 100 kb (Ca4:15,801,345..15,901,345) which overlapped with the AB4.1 region detected with GWAS (**Figure 2**). Moreover, this region was found to be under balancing selection using Tajima’s D statistic (**Figure 2**). The average nucleotide diversity (𝜃π) of AB susceptible lines across the whole genome was similar to that of the 18 AB resistant lines. The pattern of nucleotide diversity distribution was similar in the two groups except on Ca4 where almost half of the chromosome (starting from 23 to 50 Mb) showed a remarkably reduced level of nucleotide diversity in AB resistant lines compared to AB susceptible lines (1.14E-05 vs. 4.5E-05). Additionally, the extent of LD observed on chromosome 4 was 23,000 kb (*r*^2^= 0.2 cut-off) which was approximately 50 times larger than that observed on chromosome 8 (**Table 1** and Supplementary Figure S2). This indicates the occurrence of selective sweeps, possibly resulting from selection of AB resistance in the Australian chickpea breeding program.

**FIGURE 5:**
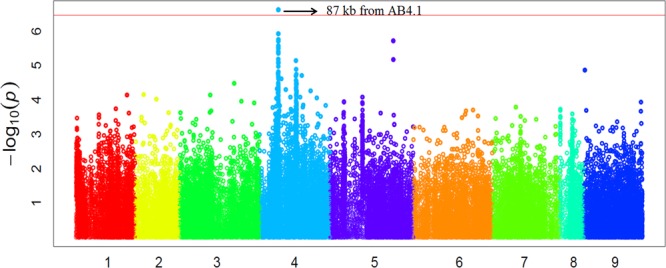
**Manhattan plot of GWAS validation using 132 advanced lines.** The physical location of SNPs are in order according to chromosome number 1 to 8 while 9 represents all unassembled contigs. The red line is the significant threshold of *p*-value = 3.47E-07, equal to a level of 0.05 after Bonferroni correction. A SNP (Ca4:15,768,013) above the threshold located approximately 87 kb from AB4.1 is indicated.

In order to validate the GWAS results based on 59 released varieties, we screened a distinct set of germplasm, comprised of 132 advanced lines for AB resistance. We observed large variation in AB resistance (*p* < 0.0001) in this germplasm, ranging from almost without damage to completely dead (Supplementary Figure S4). These 132 advanced lines were subjected to WGRS and ∼144,000 SNPs were discovered in the same manner described for the 69 genotypes. Combining SNP data with AB resistance data, GWAS identified one SNP, significantly (*p*-value = 2.40E-07) associated with AB resistance was located at a position (Ca4:15,768,013) approximately 87 kb from AB4.1 (**Figure 5**). The 20 significant SNPs present in the 59 varieties were not present in the 132 advanced lines probably due to lack of reads mapped to these 20 SNP regions. The LD surrounding the AB4.1 was very high (*r*^2^ > 0.9), thus it is very likely that the significant SNP in the validation set was linked to AB4.1.

**FIGURE 6:**
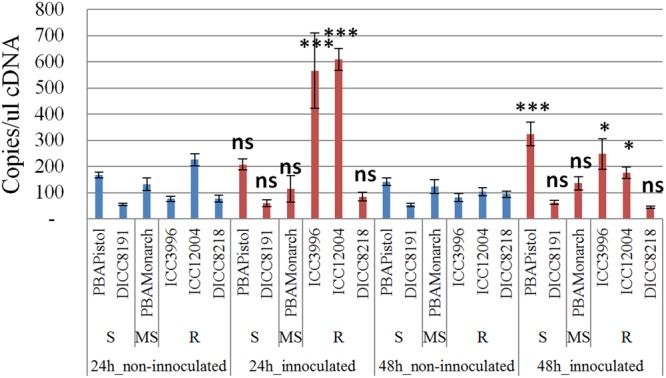
**Transcription analysis (qPCR) of predicted gene serine/threonine receptor-like kinase (Ca_05521) in AB4.1 region with six chickpea lines at two time points.** Blue: non-inoculated; red: inoculated with *Ascochyta rabiei*. S: Susceptible; MS: Medium Susceptible; R: Resistant. Significant difference between inoculated and non-inoculated lines are shown as ^∗∗∗^*p*-value <0.001, ^∗^*p*-value < 0.05. ns: non-significant

**FIGURE 7:**
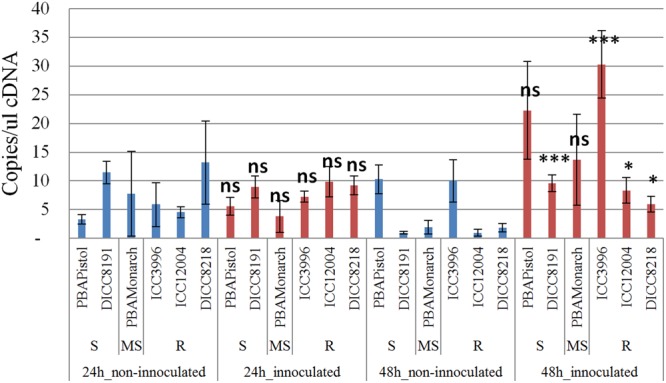
**Transcription analysis (qPCR) of predicted gene LRR receptor-like kinase (Ca_05515) located in AB4.1 region with six chickpea lines at two time points.** Blue: non-inoculated; red: inoculated with *A. rabiei*. S: Susceptible; MS: Medium Susceptible; R: Resistant. Significant difference between inoculated and non-inoculated lines are shown as ^∗∗∗^*p*-value < 0.001, ^∗^*p*-value < 0.05. ns: non-significant

In order to study the function of the 12 predicted genes located in the AB4.1 region, transcriptome analysis using qPCR was performed on six chickpea lines of differing AB resistance (PBAPistol, DICC8191, PBAMonarch, ICC3996, ICC12004, DICC8218) from the panel of 132 advanced lines. Plants were grown both with and without *A. rabiei* inoculation. Eleven of the 12 predicted genes were successfully amplified. The expression level of the 11 predicted genes was generally induced by *A. rabiei* inoculation, with some lines induced more than others (Supplementary Figures S5–S15). In some predicted genes, a change of expression generally followed the resistance level of the lines. For example, for one serine/threonine receptor-like kinase (Ca_05521), expression increased approximately 6- and 3-fold in resistant lines ICC3996 and ICC12004, respectively, 24 h post inoculation compared to mock treated plants whereas there was no significant difference in susceptible and moderately susceptible lines PBAPistol, DICC8191, and PBAMonarch (**Figure 6**). Notably, the expression level of Ca_05521 in PBAPistol became significant 48 h after inoculation while in DICC8191, and PBAMonarch the change remained not significant. Interestingly, the expression of a LRR receptor-like kinase gene (Ca_05515) was not significantly induced in all six lines 24 h after inoculation, whereas it was significantly induced in the three resistant and one susceptible line DICC8191 at 48 h after inoculation (**Figure 7**).

## Discussion

Ascochyta blight, caused by *A. rabiei* is a significant fungal disease of pulses worldwide. The outbreak of AB in Australia in the late 1990s reduced chickpea production significantly and drove a marked shift in the cultivation of chickpea from southern Australia into the northern Australian growing regions of NSW and southern Queensland. Similar to other fungal diseases, AB can be managed using different strategies which include crop rotation, pre-sowing seed fungicide treatment, in crop foliar fungicide treatment and the adoption of AB resistant or moderately resistant varieties. The latter has been a focus of chickpea breeding in Australia since the AB epidemic of the late 1990s and AB resistance is now considered an essential trait for new variety development. Genotypic analysis revealed a low level of genetic diversity among recent varieties (post 2005), an observation explained in part by the relatively narrow genetic base of breeding material in Australia. In fact, most Australian desi varieties can have part of their pedigree traced back to three Iranian landraces ICC3996, ICC14903, and ICC13729. Genetic diversity is vital to all crop improvement programs and efforts to find new sources of AB resistance and develop molecular tools to support empirical breeding is a priority for chickpea breeding in Australia. Several past studies have utilized the genetic diversity of wild species in chickpea to improve AB resistance. *C. reticulatum*, a close relative wild species of *C. arietinum*, showed much higher genetic variability compared to *C. arietinum* in this study. Sources of resistance have been found in *C. bijugum, C. echinospermum*, and *C. reticulatum* (Collard et al., 2001). However, it can be challenging to efficiently incorporate these novel sources of resistance into breeding programs; using the latest technologies such as the NGS method employed in this study can help to improve the efficiency of this process.

The genetic basis of AB resistance in chickpea has been previously investigated and QTL explaining resistance identified in bi-parental mapping populations have been reported (Pande et al., 2005; Li et al., 2015). However, the large size of the QTL regions identified (up to 30 Mb physical size) has limited their application in maker-assisted selection due to disassociation of linked markers from the resistance locus through recombination, and linkage drag which can cause unexpected genetic background effects (Mackay, 2001; Jannink, 2007; Collard and Mackill, 2008). WGRS approaches have the advantage that they can unbiasedly identify hundreds of 1000s of sequences variants (SNPs, Indels, CNVs) in a cost-effective manner. This is particularly relevant in a species with a relative small genome such as chickpea. Compared to other marker technologies such as SSRs, the mapping resolution achieved with WGRS approaches can be reached to the QTN (Quantitative Trait Nucleotide) level, which can potentially result in the detection of genetic variants in the actual gene sequence controlling a trait of interest.

In this study, we have refined the physical size of an AB resistance QTL on chromosome 4 previously identified in three independent RIL populations, to approximately 100 kb (AB4.1) and containing 12 predicted genes (**Figure 3**). The first study, using 120 RIL lines (Hadas × ICC5810) and SSRs, identified an AB resistance QTL with 14.4% explained phenotypic variation spaning around 30 Mb between marker H3C041 and TA2 (Lichtenzveig et al., 2006); The second study, using 188 F2 individuals (C 214 × ILC 3279) and 69 polymorphic SSRs, likely identified the same AB resistance QTL with 31.9% explained phenotypic variation spaning around 7 Mb between marker STMS11 and TA130 (Sabbavarapu et al., 2013); The third study, using 150 RIL lines (Lasseter × ICC3996) and 504 polymorphic SSRs and SNPs, mapped a QTL to the same AB resistance locus with 14–45% explained phenotypic variation spaning around 13 Mb between markers SSR TA146 and SNP_40000185 (Stephens et al., 2014).

In this study, 12 predicted genes were located in the AB4.1 region, including one LRR receptor-like kinase, one wall-associated kinase, one zinc finger protein, one cysteine-rich RLK and four serine/threonine RLK. The nucleotide-binding site leucine-rich repeat (NBS-LRR) family of proteins is one of the largest classes of resistance (R-genes) genes in plants with documented roles in defense signaling and pathogen recognition (Afzal et al., 2008; Ameline-Torregrosa et al., 2008; Mace et al., 2014). The LRR domain, characterized by the consensus amino acid sequence LxxLxLxxNxLxx, is likely involved in interaction of pathogen elicitor whereas the NBS region (catalytic domain) may bind and hydrolyses ATP and GTP to activate downstream phosphorylation signaling and eventually target gene expressions (Tameling et al., 2002; DeYoung and Innes, 2006). In a recent study in sorghum, it was shown that NBS-LRR genes were significantly enriched in a genomic region containing QTL for northern leaf blight disease resistance (Mace et al., 2014). A LRR receptor-like kinase (Ca_05515) was detected in the AB4.1 region under selection for AB resistance using Fst genome-scan. Using GWAS, one significant SNP (Ca4: 15,435,288) was identified to be located in the exon of this gene which led to amino acid substitution (Gly/Ala). This substitution was located in a conserved catalytic domain which has been suggested to be under purifying selection due to the functional constraints in signal transduction (Afzal et al., 2008). This catalytic domain has high similarity to the brassinosteroid (BR)-associated kinase (BAK1). BAK1 was first identified as a positive regulator in brassinosteroid signaling and later discovered to play an important role in innate immunity in plants (Chinchilla et al., 2007). Additionally, transcripts of this gene were significantly induced by *A. rabiei* inoculation in all three resistant lines. Up-regulation of RLK (including LRR receptor kinases) under biotic stress is one of the common features in early defense responses (Lehti-Shiu et al., 2009). Further research should be pursued to understand the potential role that this LRR receptor-like kinase has in AB resistance in chickpea.

Serine/threonine RLK belong to the RLK class of proteins which are involved in plant development and disease resistance via phosphorylating serine or threonine residues (Afzal et al., 2008). The structure of RLK normally includes a C-terminal intracellular kinase domain, a transmembrane domain, and a N-terminal extracellular receptor domain (De Smet et al., 2009). A recent study in Arabidopsis showed that a serine/threonine receptor-like kinase, PBL13, was involved in plant disease response by enhancing ROS burst and increasing flagellin-induced activation of MAP kinases (Lin et al., 2015). In this current work, three serine/threonine RLK were located in the 100 kb AB4.1 region. Previous studies have shown that RLK are often duplicated to accommodate disease resistance response (Shiu et al., 2004). Duplicated receptor protein kinases might be retained due to their diverse specificity in recognizing different pathogens or elicitors (Shiu and Bleecker, 2003).

A predicted cysteine-rich receptor-like kinase gene (CRK) was detected in the AB4.1 region under selection for AB resistance. CRK is a sub-family of plant RLKs with one or several repeats of unknown functional domains (DUF26) consisting of a C-X8-C-X2-C motif (Chen, 2001; Bourdais et al., 2015). Previous studies suggested that CRK was involved in biotic and abiotic stresses response; Overexpression of CRK in Arabidopsis led to hypersensitive response-like cell death (Chen et al., 2003, 2004) and increased tolerance to the pathogen *Pseudomonas syringae pv. Tomato* (Acharya et al., 2007). A large-scale study using 82 CRKs T-DNA insertion lines demonstrated that CRKs played an important role in regulating reactive oxygen species (ROS)-related stress responses such as stomatal closure caused by pathogen and abiotic factors (Bourdais et al., 2015). However, transcription level of this CRK gene was not correlated with the AB resistance ranking of the six lines examined indicating this gene might not be involved in AB resistance.

Wall associated-receptor kinases (WAK) are another sub-family of plant RLKs with epidermal growth factor (EGF) repeats in the extracellular domain that can bind to pectin generated by invading pathogens (Kohorn and Kohorn, 2012; Kohorn, 2016). Two recent studies in maize showed that WAK played an important role in response to head smut soil-borne disease caused by fungus *Sporisorium reilianum* (Zuo et al., 2015) and northern corn leaf blight caused by fungus *Exserohilum turcicum* (Hurni et al., 2015). One WAK gene was detected in the AB4.1 region under selection for AB resistance. Transcripts of this gene were significantly induced by *A. rabiei* inoculation in two resistant lines ICC3996 and ICC12004 but not in another resistant line DICC8218. A possible reason may be that DICC8218 has a different resistance mechanism that does not involve this particular gene, or that this gene is not involved in AB resistance.

To dissect genetic variation of genomes, many whole genome resequencing projects have been carried out in human (Abecasis et al., 2012; Auton et al., 2015), livestock (Daetwyler et al., 2014; Ai et al., 2015), and plant species using NGS technology (Huang et al., 2012; Varshney et al., 2013; Li et al., 2014). Although numerous genomes have been sequenced and hundreds of thousands of markers discovered, this information could not be transferred into breeding without high-throughput and accurate phenotyping technology. For complex traits, such as yield and drought tolerance controlled by numerous genes with small effect size and highly influenced by environment, a large number of samples precisely tested in different environments are needed to secure enough statistical power to discover marker-trait association. For simple traits with high heritability such as some disease resistance traits controlled by a few major genes with large effect size, a relatively small sample can have enough statistical power as demonstrated in wheat (Jordan et al., 2015) and Arabidopsis (Atwell et al., 2010). As shown in this study using only 59 genotypes yet with a large amount of maker information (∼250,000 SNPs), we have narrowed down a major AB resistance QTL interval (up to 30 Mb) to a 100 kb region containing only 12 predicted genes. Additionally, we have validated this result with a larger sample size.

## Summary

Both natural and artificial selection processes have marked the chickpea genome with various selection signatures. One common signature is a selective sweep, characterized by an extensive genomic region with a decreased level of genetic diversity. The analytical power to discover these signatures has been improved using NGS technology and advances in statistical methods. By resequencing 69 diverse chickpea genotypes, we have detected a 100 kb genomic region containing 12 predicted genes under selection for AB resistance using GWAS and Fst genome-scan. A subsequent GWAS validation study has confirmed this finding. Transcriptional analysis using qPCR has shown that some predicted genes were significantly induced in resistant lines after inoculation compared to non-inoculated plants.

## Author Contributions

YL conceived, designed, and interpret the study. YL and PR analyzed the data. JB contributed to sequencing; KH and JD contributed to phenotyping; DE supervised data analysis; TS supervised the study and edited the manuscript; All authors read and approved the manuscript.

## Conflict of Interest Statement

The authors declare that the research was conducted in the absence of any commercial or financial relationships that could be construed as a potential conflict of interest.

## Footnotes

1 http://www.genome.jp/kegg/
