# Supplementary material for: Genome Analysis Identified Novel Candidate Genes for Ascochyta Blight Resistance in Chickpea Using Whole Genome Re-sequencing Data
Source: Front Plant Sci. 2017 Mar 17;8:359. doi: 10.3389/fpls.2017.00359 (PMC5355423; doi:10.3389/fpls.2017.00359)
Supplement: Supplementary file 1 [file Data_Sheet_1.pdf]

Supplementary Material

Genome analysis identified novel candidate genes for ascochyta blight resistance in chickpea using whole genome re-sequencing data

Yongle Li, Pradeep Ruperao, Jacqueline Batley, David Edwards, Jenny Davidson, Kristy Hobson, Tim Sutton*

*** Correspondence:** Corresponding Author: Tim.Sutton@sa.gov.au

Supplementary Table 1, Material used in this study

Name	Type	Release date	AB category	Attributes	Mapped seq.depth
Tyson	Desi	1978	ABS	Wide adaptation	2.28
Opal	Kabuli	1979	ABS	NA	2.27
Macarena	Kabuli	1984	ABS	Large seed	2.14
Amethyst	Desi	1987	ABS	Harvestability	2.17
Garnet	Kabuli	1987	ABS	Large seed	2.36
Dooen	Desi	1988	ABS	Yield	2.18
Kaniva	Kabuli	1988	ABS	Yield	2.72
Narayen	Kabuli	1989	ABS	Flour yield	2.17
Semsen	Desi	1989	ABS	Large seed	2.71
Barwon	Desi	1991	ABS	PRR resistance	2.65
Norwin	Desi	1992	ABS	PRR resistance	2.73
Desavic	Desi	1993	ABS	Yield	1.79
Lasseter	Desi	1996	ABS	Yield, large seed	2.49
Bumper	Kabuli	1997	ABS	Large seed	2.81
Gully	Desi	1997	ABS	Virus resistance	2.19
Heera	Desi	1997	ABS	Yield	2.14
Sona	Desi	1997	ABS	Yield	2.41
Jimbour	Desi	2000	ABS	PRR resistance	1.87
Howzat	Desi	2001	ABS	Yield,w ide adaptation	2.36
Moti	Desi	2003	ABS	Yield, earliness	1.66
KimberleyLarge	Kabuli	2004	ABS	Yield, large seed	2.18
Rupali	Desi	2004	ABMS	Chilling tolerance	1.73
Sonali	Desi	2004	ABMS	Chilling tolerance, FW	2.02
SONALI_A	Desi	2004	ABMS	Chilling tolerance, FW	1.73
Almaz	Kabuli	2005	ABMR	Yield	2.36
Flipper	Desi	2005	ABR	ABR	2.13
Genesis090	Kabuli	2005	ABR	Yield, ABR	1.98
Genesis509	Desi	2005	ABR	Yield, wide adaptation, ABR	2.43
Genesis510	Desi	2005	ABR	Yield, wide adaptation, ABR	2.37
Genesis836	Desi	2005	ABMS	FW	2.25
Kyabra	Desi	2005	ABS	Yield, PRR resistance	2.14
Nafice	Kabuli	2005	ABMR	Yield, large seed	2.51
Yorker	Desi	2005	ABMS	PRR resistance	2.12
YORKER_A	Desi	2005	ABMS	PRR resistance	1.74

Genesis079	Kabuli	2009	ABMR	Yield, wide adaptation	2.24
PBAHattrick	Desi	2009	ABMR	ABMR, PRR resistance	2.29
PBASlasher	Desi	2009	ABR	Yield, ABR, seed quality	1.89
Genesis114	Kabuli	2010	ABMR/ MS	Yield, medium to large seed	2.28
GENESIS114_A	Kabuli	2010	ABMR/ MS	Yield, medium to large seed	1.49
GenesisKalkee	Kabuli	2011	ABS	Yield, large seed	2.37
PBABoundary	Desi	2011	ABMR	Yield, wide adaptation, ABMR	2.58
PBAPistol	Desi	2011	ABS	Yield, early flowering	2.28
Ambar	Desi	2012	ABR	ABR, early flowering	2.76
Neelam	Desi	2012	ABR	ABR, wide adaptation	2.91
PBAStriker	Desi	2012	ABMR	Yield in short seasons, ABMR	2.13
PBAMaiden	Desi	2013	ABR	Large seed	2.73
PBAMonarch	Kabuli	2013	ABMS	Early flowering	2.68
PBASeamer(CIC A912)	Desi	2016	ABR	PRR resistance, ABR	2.24
AB001	Desi	NA	NA	NA	0.65
AB002	Desi	NA	ABR	NA	5.65
AB015	Desi	NA	NA	NA	7.76
AB019	Desi	NA	NA	NA	10.37
AB022	Desi	NA	ABMR	NA	1.90
AB023	Desi	NA	ABR	NA	2.45
AB024	Desi	NA	NA	NA	8.04
AB034	Desi	NA	NA	NA	7.20
DICC8022	Desi	NA	ABMR	NA	5.10
DICC8174	Desi	NA	ABMR	NA	5.48
DICC8196	Desi	NA	ABR	NA	5.86
DICC8212	Desi	NA	ABMR	NA	7.06
DICC8220	Desi	NA	ABR	NA	7.46
DICC8296	Desi	NA	ABR	NA	5.41
DICC8310	Pea	NA	ABR	NA	6.16
ICC1882	Desi	NA	NA	NA	4.54
ICC283	Desi	NA	NA	NA	3.54
ICC4958	Desi	NA	NA	NA	4.03
ICC8261	Kabuli	NA	NA	NA	4.52
PI489777	C.reticul atum	NA	NA	NA	3.52
WACPE2160	Desi	NA	ABR	NA	8.09

ABS: ascochyta blight susceptible. ABMS: ascochyta blight medium susceptible. ABMR: ascochyta blight medium resistance. ABR: ascochyta blight resistance. PRR: Phytophthora root rod resistance. FW: Fusarium wilt

Supplementary Table 2, Primer sequences for qPCR

Gene ID	Sense Primer	Anti-sense Primer
Ca_05511	GCTGCTCCTCCTCCCTTC	TCGTTGACAATGCGGATGAC
Ca_05512	AGAGAAGAGAAGGCAGTATGACAG	GGCTGAGATGGTAGTCTTGATAGG
Ca_05513	GTCTTTATCACCCATCATCAC	CAGCAACCGAGGTATATCC
Ca_05514	CTTCATTACCAAGAACACCAC	AACAATTCCTCATCATCCATATC
Ca_05515	CTGAAGCAATAGACGGTAG	CAGCAGTCCATTCCTCTC
Ca_05516	CAAGATGGAAGTCTCGTGGCAGTG	TTCCTCGCCGTTCCCGTTCG
Ca_05517	TGACAGTGGTGAGTTTATGGTG	GAAAGGGTTTGGGCGGTATC
Ca_05520	GGAGAGTAGAACAGTTTG	ATGTAGATAAGCCAATGC
Ca_05521	GTTGCCTTAGAGACTGCTAC	ACCTGGTGTTCCTTGTGG
Ca_05522	CGAGATGGACGAGAAGTTG	TATTCATACACAAGCAGTAGTTC
Ca_05523	ACGAACACAATTACAGGAGAG	GGCAGTAGCAGTTTCTATGG

Supplementary Table 3, Percentage of genome under selection

Chromosome	Total length (kb)	D>2 (kb)	% D>2	D<-2 (kb)	% D<-2
Ca1	49000	5500	11.22449	500	1.020408
Ca2	37000	1600	4.324324	100	0.27027
Ca3	67000	1200	1.791045	300	0.447761
Ca4	59000	6500	11.01695	300	0.508475
Ca5	69000	100	0.144928	100	0.144928
Ca6	67000	1500	2.238806	1000	1.492537
Ca7	56000	2400	4.285714	0	0
Ca8	20000	1300	6.5	500	2.5
total	424000	20100	4.740566	2800	0.660377

Supplementary Table 4, Predicted genes located on chromosome 4 (Ca4: 15,855,018..15,980,584)

Kabuli reference genome 1.0	NCBI LOC	Annotation
Ca_05523	LOC101515334	serine/threonine protein kinase
Ca_05522	LOC101488413	serine/threonine protein kinase
Ca_05521	LOC101488744	serine/threonine protein kinase
Ca_05520	LOC101489079	wall-associated kinase
Ca_05519	LOC101489412	uncharacterized protein
Ca_05518	LOC101489732	uncharacterized protein
Ca_05517	LOC101490487	serine/threonine protein kinase
Ca_05516	LOC100785979	cysteine-rich receptor-like kinase
Ca_05515	LOC105851082	LRR receptor-like kinase
Ca_05514	LOC101490811	uncharacterized protein
Ca_05513	LOC101491135	uncharacterized protein
Ca_05512	LOC101491667	uncharacterized protein
Ca_05511	LOC101491977	zinc finger protein

Supplementary Table 5, Information of the 21 SNPs significantly associated with AB resistance

SNP	Chr.	Position_Ref 1.0	P.value	Upstream 100bp sequence	Downstream 100bp sequence
Ca00000335415	4	15961111	1.46E-05	ATCCTTTTGAGTTTATTTTCTCACACG CCTTGACAAGCAAGTGACCAATTACT GAATTTTAACCAGCATATTTAAGTTG AACCATCTTTTTCATTTATATG	GTGTAAGCTTGATTAATCCAATTAGA GAAATGTACAAGTTTTAACATAAAGA AATTTAGCCCTTCTCAAGCATGTTGGG ACCAGGGAGTTTAATTTGTTCC
Ca00000335663	4	16027381	1.67E-05	ACCCATGGCGCAGCACGGTAACAAA CAACCTCAAACACAATTAATTAGATG TTTAATTATGTCTCAATTCAATTTGTT TTTTTTGTTGTTGTTGTTGTTGC	CTGCAAATTGTAACTATTTGTCTTGGT TAATTTTAAAGTCCATTTTCTAATGCA TTGCATATATAGAAATTGGTTTTGTTT AATTTTGGCTTTTTGCGTTG
Ca00000335607	4	15996386	1.98E-05	TGCGCCTTGAAGATCTTAGATGTGAA ATTGAATATTTGTTTGTTGATGATTTT GACTGTGAAATTCAACATTTGAGCCT CGATTACTCTCTTAAGTGTGAA	AAATTTGGTATATAAATTTTCCTTAGA ATGATGCTTGTTTGAATGTTAAACAAG TGATTTTGAAAGTAAATTGTCCCTTTT GATTTCGAGAAGGTCTACGA
Ca00000335608	4	15996485	2.21E-05	AAAATTTGGTATATAAATTTTCCTTA GAATGATGCTTGTTTGAATGTTAAAC AAGTGATTTTGAAAGTAAATTGTCCC TTTTGATTTCGAGAAGGTCTACG	GAGATGACAAACAAAAATTTTCAAAA GATTTTTTTGAAAACTATAATGATGGA AATCAGAAAGTTTGTTGGAAATTTCTA CCGTGGTTGAACAAGATATTT
Ca00000335697	4	16031842	2.25E-05	CTAAGTAACGTTTTAATTAATAATAT GAAGACTAATTTAAATTTGTAACTTT GTCTAGAACTAAAGCTATGGATATTT TCAAAACTAAACTTGGTCTAATC	CTATCTAATCATTTTTCCTTGAATTAA TAATCACCTTCACATGATATTATAATA CATACATACATGAACTAAAATCATAG GATTCGATCTTAAAATGAAAT

Ca00000335831	4	16052515	2.30E-05	TTATGAATAGAAATAACACAAATTGC TTGAATATTCGAGAGATCTTGGATAT CTCCCAAATTCTTATTCCAACAATATC TCTCAATCCCCTATTACTATTG	GATCAACAGGCCTTATTGATCTCTTCT AAATATCTCAGCCTAAATATTTGGAG TTATAATACTCCCCCCAACCCCCTGAA CATTCACCTTGTCCTTAAGGC
Ca00000335819	4	16050152	2.34E-05	TATCTACCCAAGTTATGATTATGCTC ACTGCATTGTGGATTCGCTTGAGAAT ATCACACACTCAGTAAGTTTCATCCA CTAAATTGTGTTATATTAAAAAT	TAATGATTTTTGTAGATATTTCCCCTT TGACAAAGTTGATAATGCCTGGATAC CTCAAGTCTGCTGGACGTCTAAATTCT CTTTCTGCATTGATATATTTA
Ca00000335482	4	15972941	2.49E-05	CATTTCATACACACTGGCAGTCATTTT GTTACAACATATGGAATTTCATTAAT GTTATTTCATAAAATTCAAATCAAAT CTCAACTGTTGCGAGTATTGTT	TGCCAGGAGCTTTTGTGTTTCTTGTAA AAATGGGGATTTAAAATTCACGGTTC AGTGCTGAAATATGAATTTAAAATAT AAAATTGATGTGTGAGTTTCTT
Ca00000335321	4	15943658	2.56E-05	CTTCAACCTTGTGAAGTCTCAGAACT AAAGCCTGGCACACATATATTTGCTG TTTATGGTCTGAATAATTTTTTTGCTT CATTGTTCTTTGGCTTGTTTGG	GGATGTTTTTTCACGTAAACTAATTCA AGTTGATATTGTTTAGGTGATAACTTC TTCAAGACTGCTAGCTATACAATTGA AGCAGTTTGTGCGAAATCACA
Ca00000335478	4	15972622	2.56E-05	ATAGCATAAACATAGCAATAATGAAT GGCTTATTTTGAAAATTAAAACCACA TAAGAAAAGTTCGAGTTAATATGAAA ACTTTAGGAGTGAAAACTTCTCA	ATTGTAAGAATTATACCAAACAGATA GGTTTCGGAGCAACAGAAAATCCAAT TAAATACAATCCTCCACCAGACATTTC AATAACTTTGACCAACCAATCC
Ca00000335820	4	16050372	2.60E-05	TTTTAAATTATATCAATCATCCCGAAT ATATTTGATATAATGTGTACTTTTGGG CAGTATACATTGTACTATTCATTATA GCAGTTGGAGTTTTGTTGCTG	GCGATAAGTGAGCTATGTACAATTCA GTGATGTAATGGTTTTCATATAGTATT TTATGCACAACACTCCATAGCAAATT GGCTCTGTTTTTTTTTTTTTTG

Ca00000335315	4	15942039	2.66E-05	TACAGTCAGACCCCTTCCAATTGGAA<br>CATCAGTTAGTGGAAAGGTTCTCACC<br>TTCTACACGATGAATGAAGCAATACA<br>GTAATCTTTCTGGTGCCTATTTA	ATTCTTGTTCAACTTATGTGACAATTT<br>GTTGCAGGTAGAGAAGCAATGTGCTC<br>ATTTTTTTGGTGTGACAATAAATGAGC<br>AGCAAGCTGAGTCAGGGATTG
Ca00000335316	4	15942209	2.70E-05	ATAAATGAGCAGCAAGCTGAGTCAG<br>GGATTGTAGTAAGAGTTACTTCAACT<br>GCACAAAGCAAATTCAAGGTATCAAT<br>GACGCCTTTTTTACAAATATTCCT	TCTGTCTTTGTCTCTTTGTCTGTTGCAT<br>TTTTGCTTCCGGTTAATATGATGTTGG<br>ATTATAATATTGAAGGCTGATTTTTCA<br>GTTGCTCTATTTCGAACAA
Ca00000335325	4	15944195	2.70E-05	TCTGCTCCATTAAGTTGTAGCAAGCT<br>GGATCAGTTGCAGGTTTTGCTCACCT<br>GGCTTACATGACTGGCCGATAATCTG<br>TAGCAATCACGTATTATAATTTG	GACATTTGATAGAGCAAAAGGAAACA<br>ACTCAGCTGTCGAGCCATACCTTATTT<br>TGGCCTTTAATATACATTGTTATGTGA<br>ATATGTTGTACATAATGTCAA
Ca00000335829	4	16051721	2.70E-05	CTAGCTTTGATACCTAGCTATATATTC<br>TTGTAGACTTGTATCATTCATACAAC<br>AATCATACTCAGAATATTTAGTTCGG<br>TTTCTATCTTCATTTTCTCTTA	ATTTCTGAAACATAATAATGAAGTAA<br>AATTAAAATACTGACAGGTGCCTTTTC<br>CAAATTAATTATTTCACTGTTGACAAT<br>GTGTTCTGTTTGCATAAGTTT
Ca00000335817	4	16049395	2.77E-05	GTGGATGGGCTGGAAACCATTCAAGG<br>TATGTTTGTTTGCACCTCTTTTGTTGA<br>TTGTGTGGAAATTGTCCATCCTAGCTT<br>TTTAGATGTTTAACAGCTTTA	ACACTTGTGCAGATTACTTACACAAG<br>CGATTATTTCCAAGAACTATATGAATT<br>AGCCGTGGAGCTCATTAGAAGAGGTT<br>GTGCTTACGTTGATCATCAGGT
Ca00000335672	4	16029163	2.90E-05	AAGCGTCGAACACTAAGTAATATTCG<br>AATTCAGATTCATAACATTGTGAGAG<br>TCTAACTTATTCTACTAGACTCAATTT<br>CTGATAGTCTCTATTTCTATCA	ATATTAACAACATATTATAATTCAAA<br>GCTTATATTTTGAAATTTCCATCATTG<br>GACATGTTATAAGGCAGTACTAGACT<br>ATCATAATTCAAAACTGATGTT

Ca00000335172	4	15925020	2.93E-05	ATAGACGGTAGAAAACAATCACAAA AATCGGACGTGTACTCTTTTGGTGTTC TGTTGCTAGAGGTTCTGACAGGGAAG TGTCCCTCTGCTGTGGACAATGG	GTGGAAGTGGTTATGGTGGGGGGGTG ATGGATCTTCCAAGGTGGGTGCAATC AGTAGTTAGAGAGGAATGGACTGCTG AAGTATTTGATTTAGAGTTGATG
Ca00000335193	4	15929393	2.99E-05	GGTTTTAGAGCAAAATATCTTTAAAT TAAATTTTGAATTTTTTTTATAAATTA AAAAATAAAATAATTAAATTATACTG AAAAAAGTATGCAAAATAAAAT	TTATAAATTAATTAAAAAAATTGAGA TTGTTGGTGAATAGAATCAAAGCAGA TGATATAATTTGTTGAGATATTTTTGT TACATAAAAATAAAATAAAAAA
Ca00000335488	4	15973984	3.23E-05	AGAGGATATCAAAAGAGGCGGCGGC ACGTGCGTGACCGAGATGACTGTAGT CATAGGCAGTGACTCCACAAACATAC ATAGAAACCTTGCCAGGTTCTATG	GGTTTTGAAAATGTCTTTCTGCTGAGT CAAGGAATTGTAAATCTTCAACTCAA CTCGTTCCTCCGCCATTGCTGCCACTG TCTGCACACAAACGCGTTACA
Ca00000120693	4	15768013	2.40E-07	TTTTGTACAAGAGAAAATGTAGGTAT TTACAACTTTTTTTCATATAGATAAGT TATTTTCGTCCCTTTATATGAACTGAG AATTTTTTATCTTGAGTGTAA	AGATACAGACTAAGTTAGGCCTAATT TTTTTTGGGTTTATTTTCTTCATTTACA AAAAGTTGGGCCCAAATTTTTTTCTGA TGATGAAAGGGCCGAACATGAAC

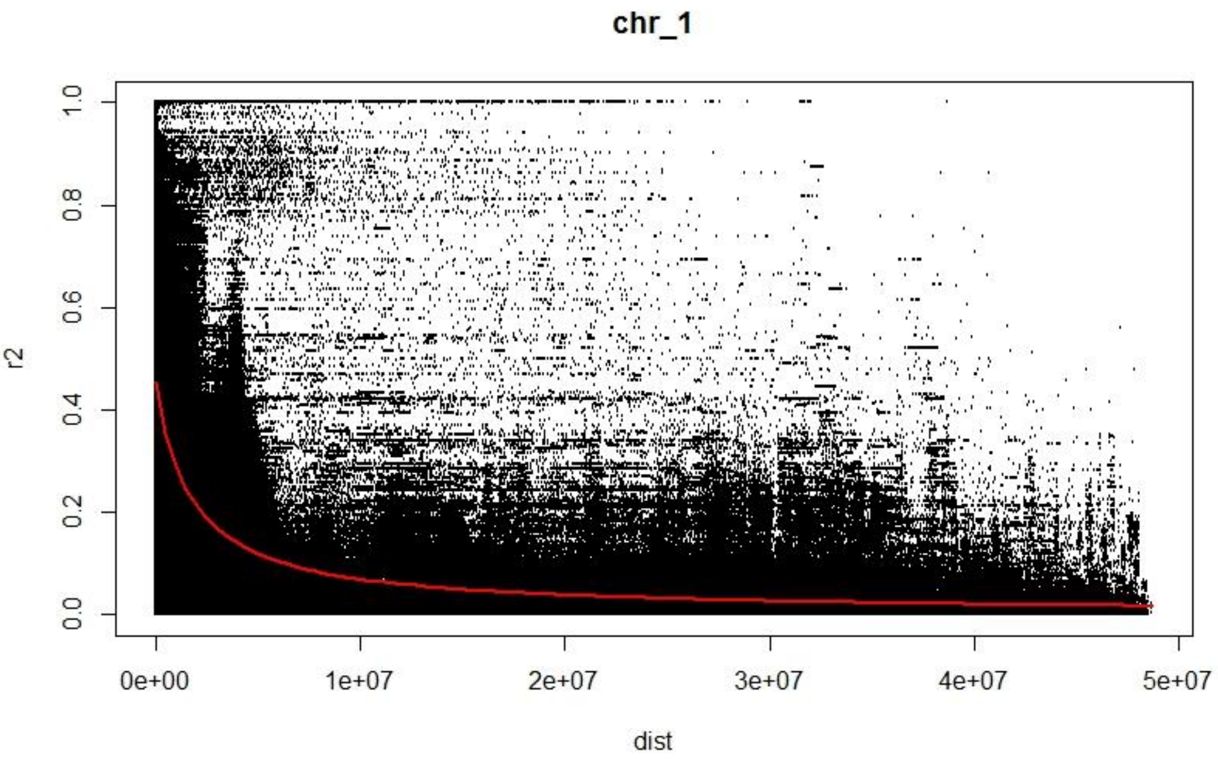

Supplementary Figure 1a **Pair wise LD *vs* physical distance in chromosome 1.**

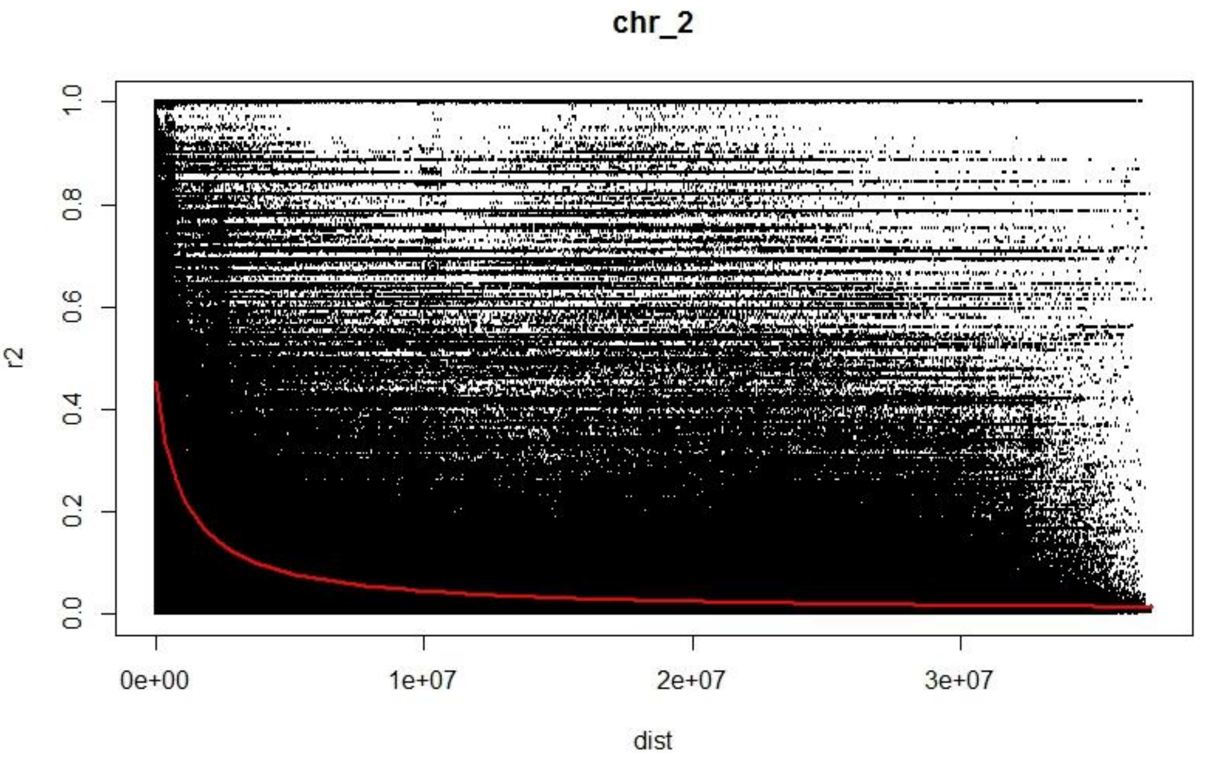

Supplementary Figure 1b **Pair wise LD *vs* physical distance in chromosome 2.**

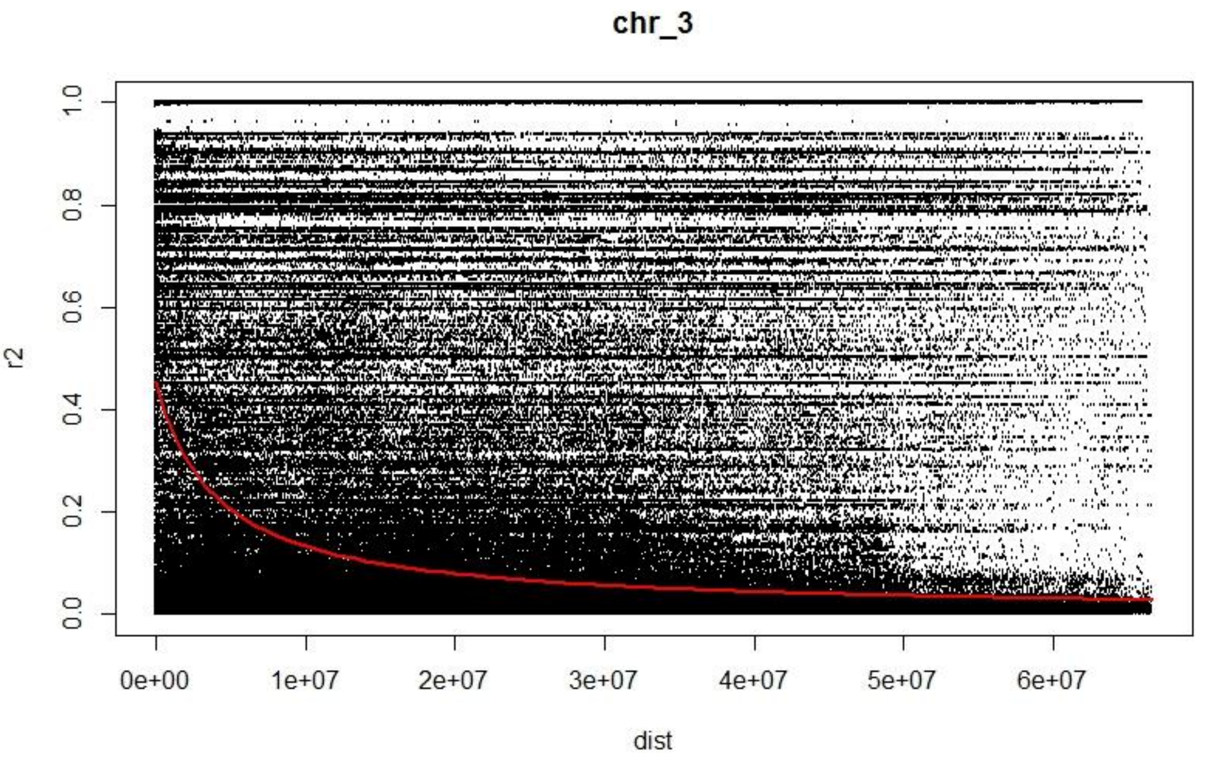

Supplementary Figure 1c **Pair wise LD *vs* physical distance in chromosome 3.**

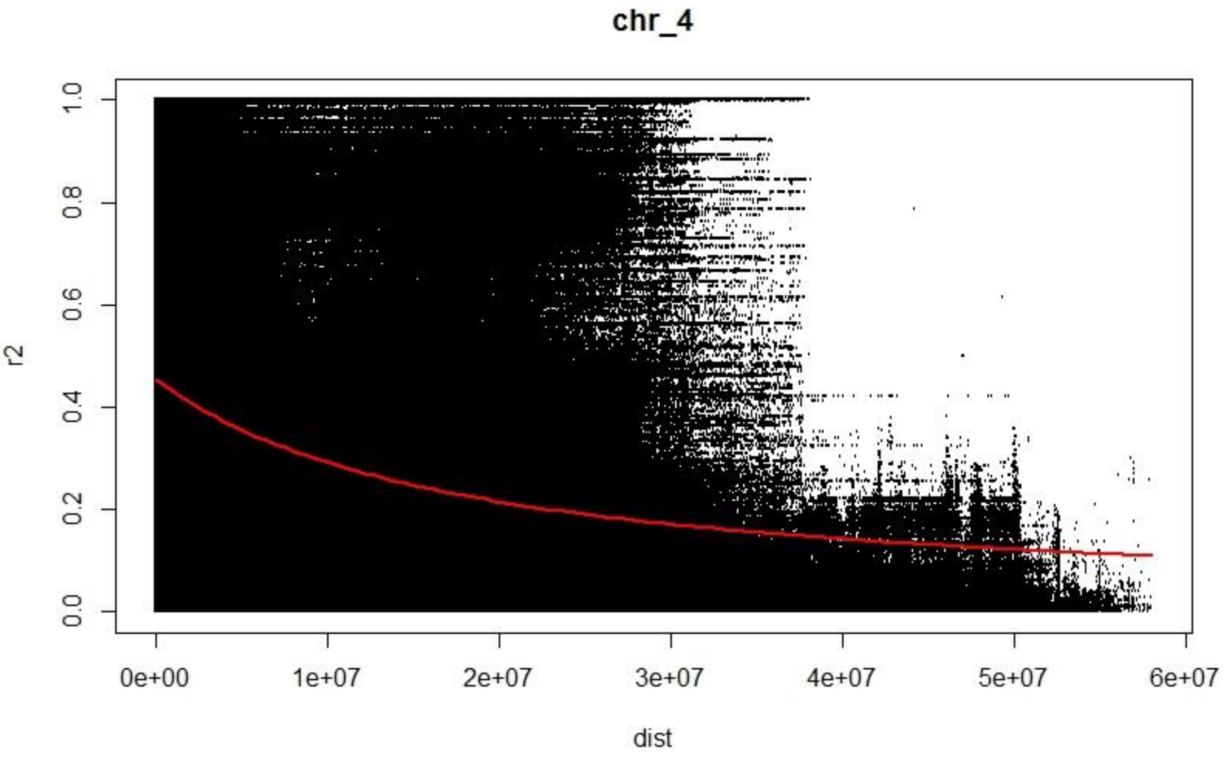

Supplementary Figure 1d **Pair wise LD *vs* physical distance in chromosome 4.**

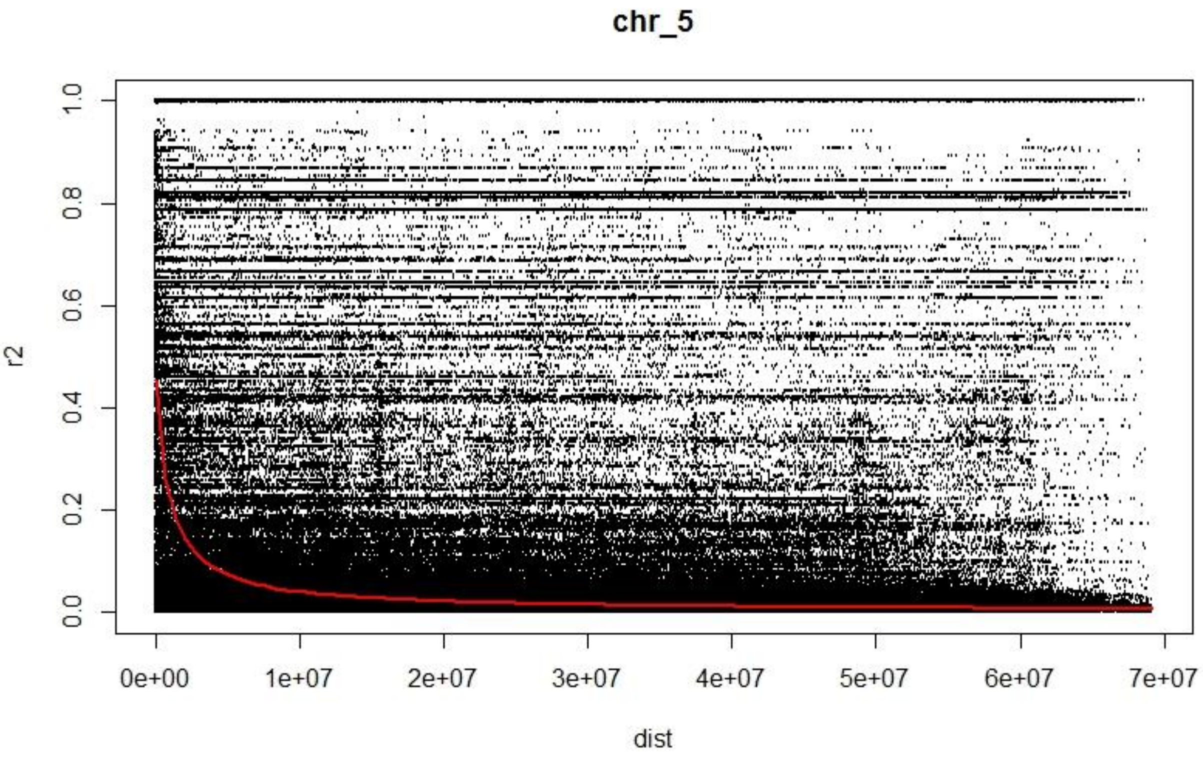

Supplementary Figure 1e **Pair wise LD *vs* physical distance in chromosome 5.**

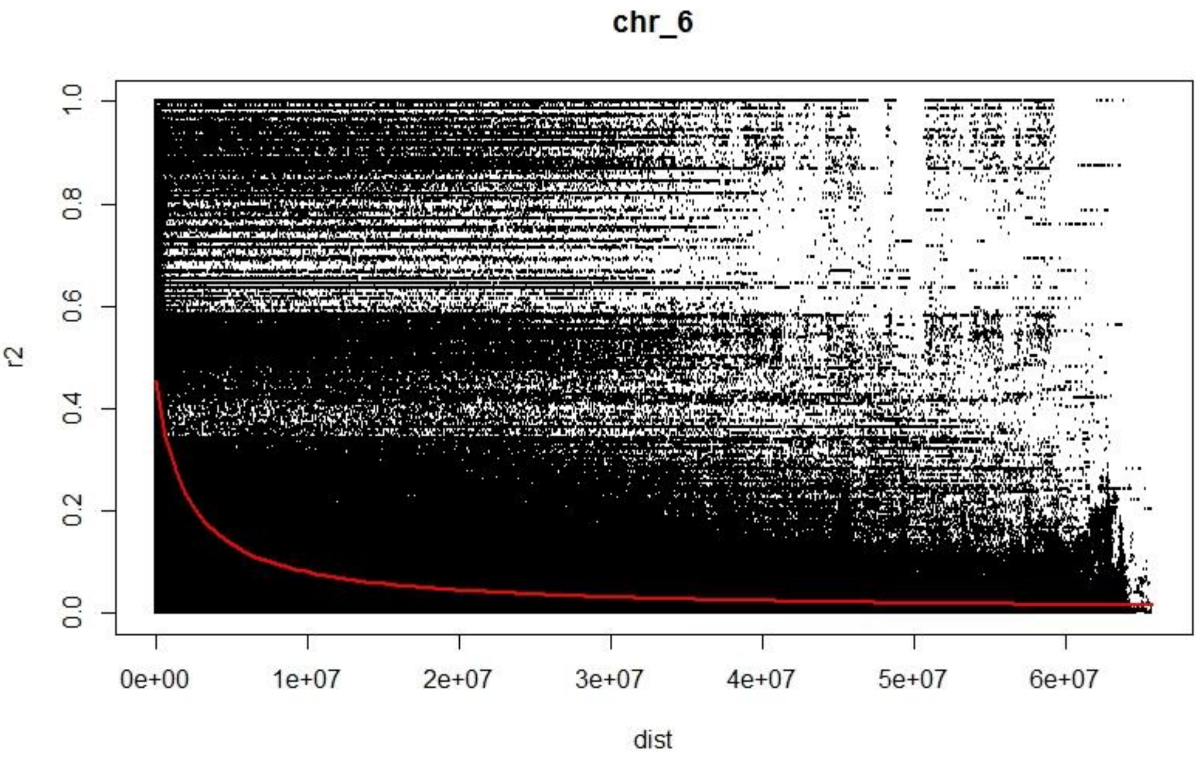

Supplementary Figure 1f **Pair wise LD *vs* physical distance in chromosome 6.**

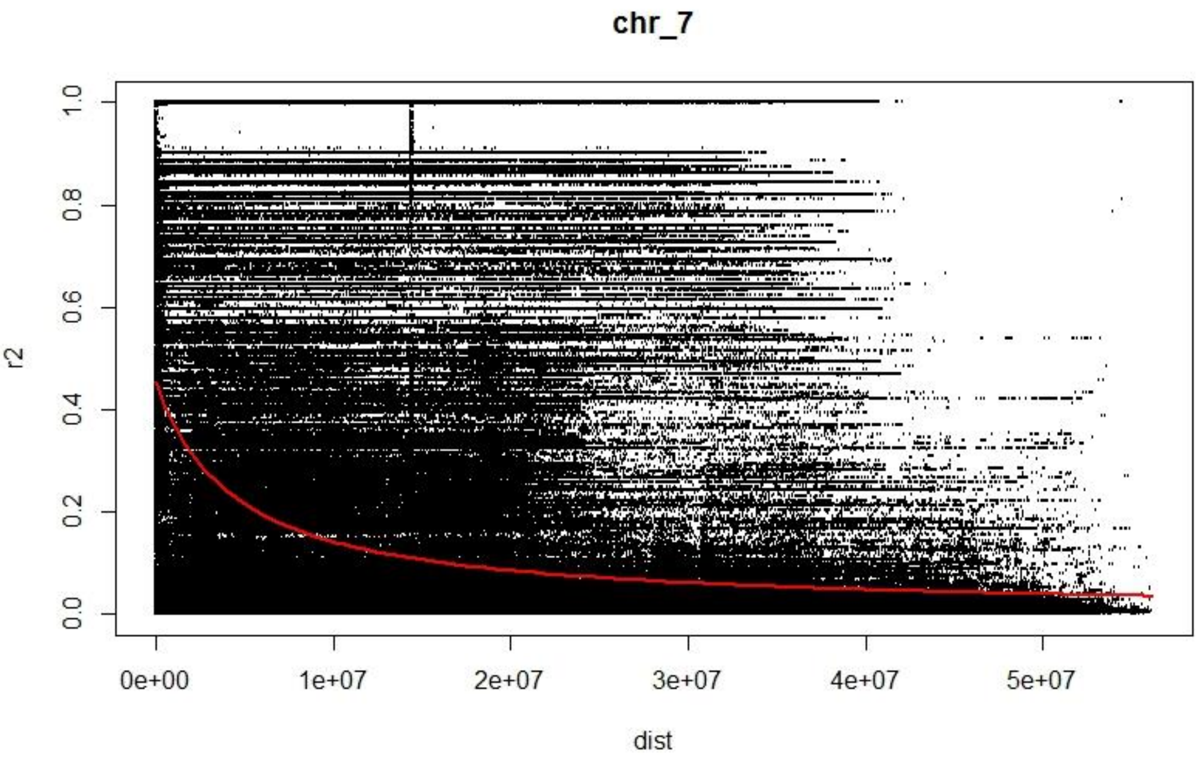

Supplementary Figure 1g **Pair wise LD *vs* physical distance in chromosome 7.**

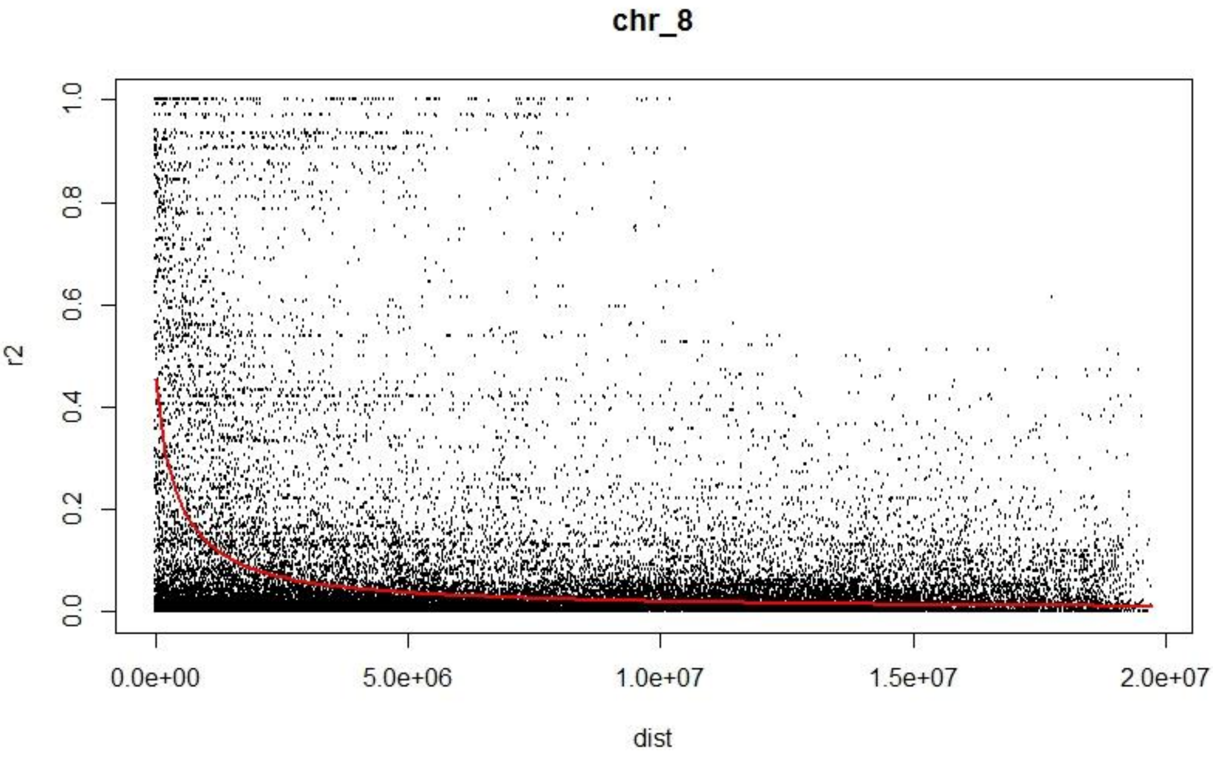

Supplementary Figure 1h **Pair wise LD *vs* physical distance in chromosome 8.**

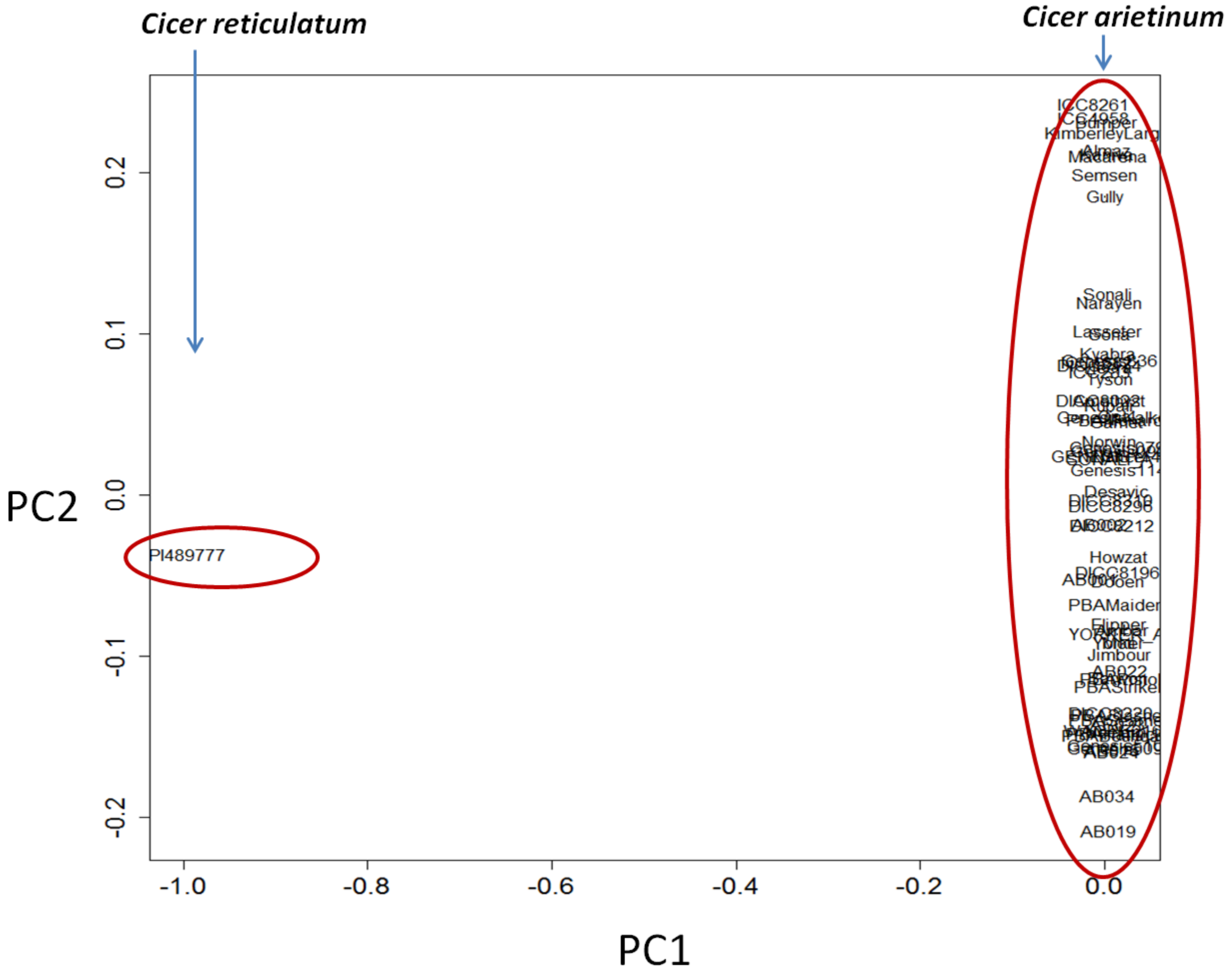

Supplementary Figure 2 **The first two PCs in principle component analysis of the 69 chickpea genotypes based on SNPs data**

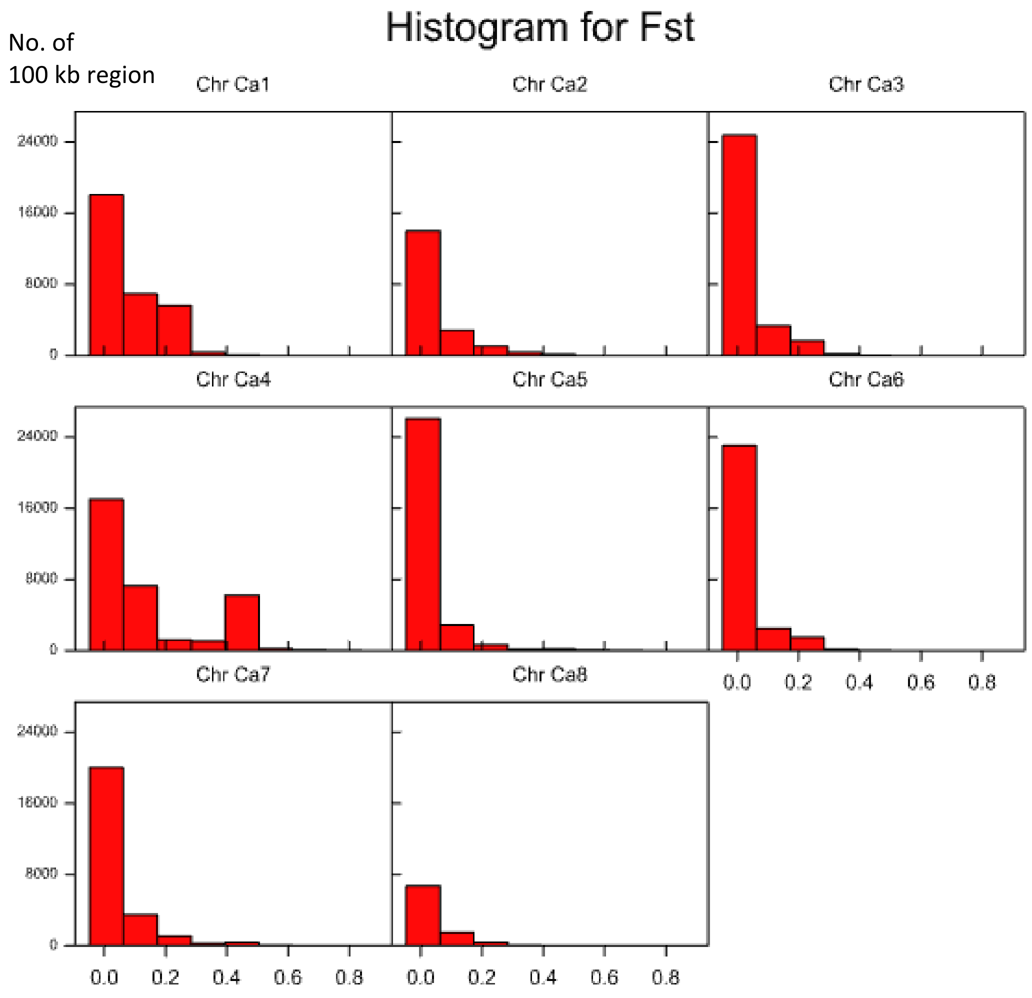

Supplementary Figure 3 **The distribution of Fst value in the eight chickpea chromosomes**

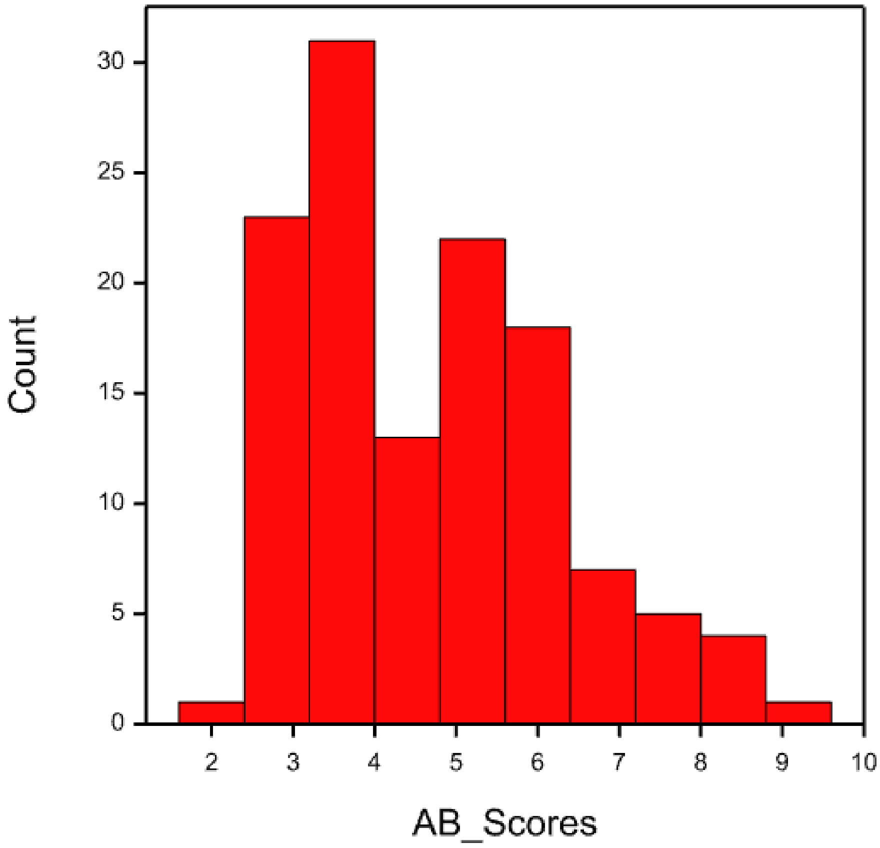

Supplementary Figure 4 **The distribution of AB scores in the validation set**

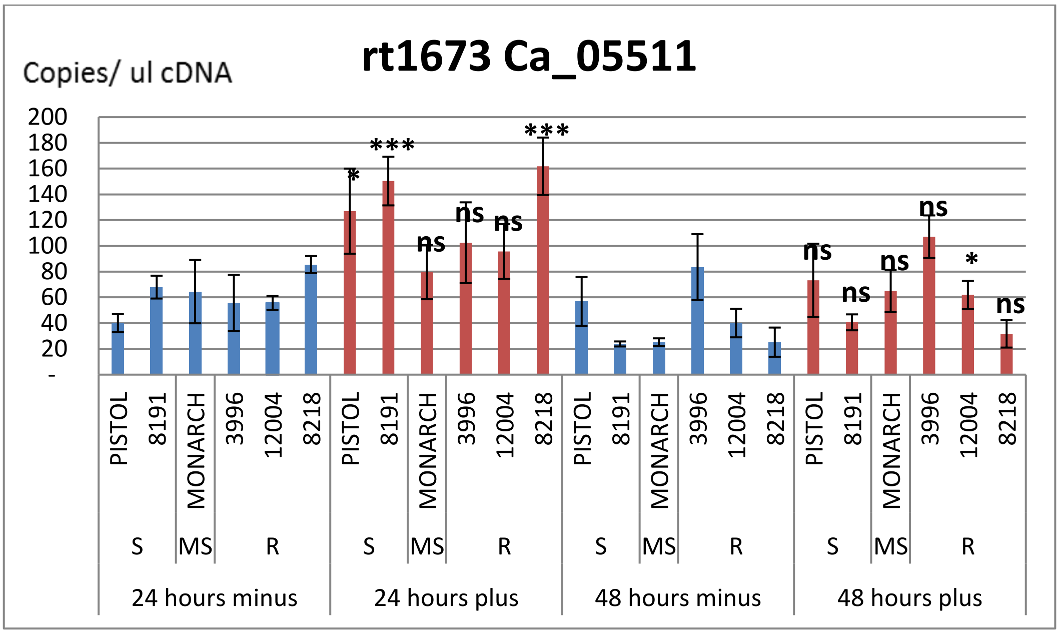

Supplementary Figure 5 **Transcriptional analysis (qPCR) of predicted gene encoding zinc finger protein (Ca_05511) in AB4.1 region with 6 chickpea lines at two time points.** Blue: non-inoculated; red: inoculated with *A rabiei*. S: Susceptible; MS: Medium Susceptible; R: Resistant. Significant difference between inoculated and non-inoculated lines are shown as *** (p-value<0.001), * (p-value<0.05). ns: non-significant

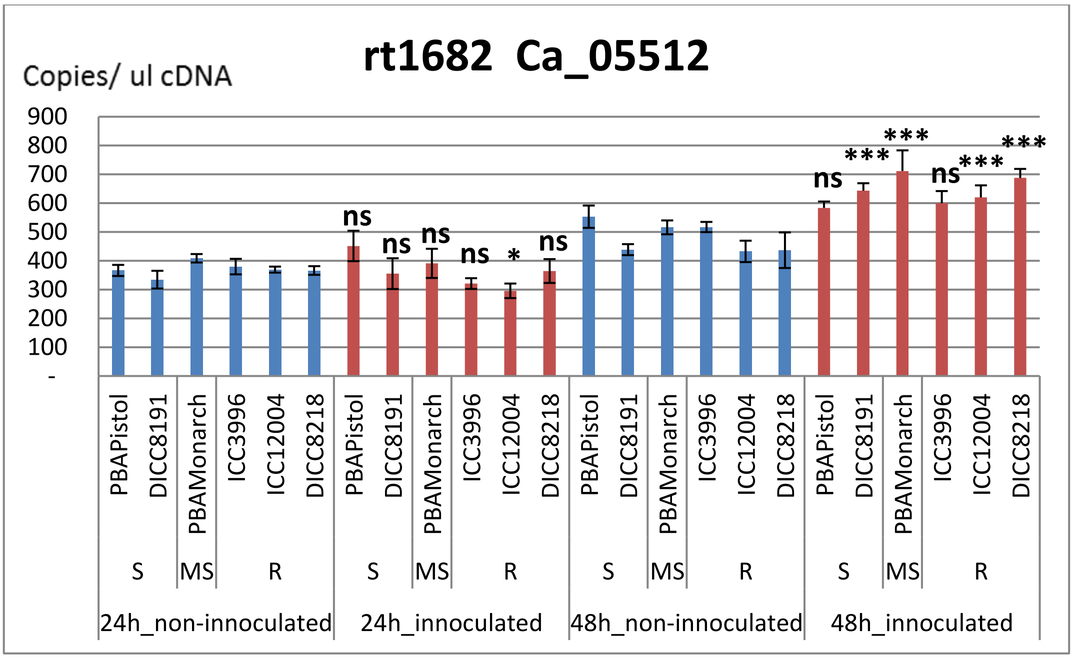

Supplementary Figure 6 **Transcriptional analysis (qPCR) of unknown gene (Ca_05512) in AB4.1 region with 6 chickpea lines at two time points.** Blue: non-inoculated; red: inoculated with *A rabiei*. S: Susceptible; MS: Medium Susceptible; R: Resistant. Significant difference between inoculated and non-inoculated lines are shown as *** (p-value<0.001), * (p-value<0.05). ns: non-significant

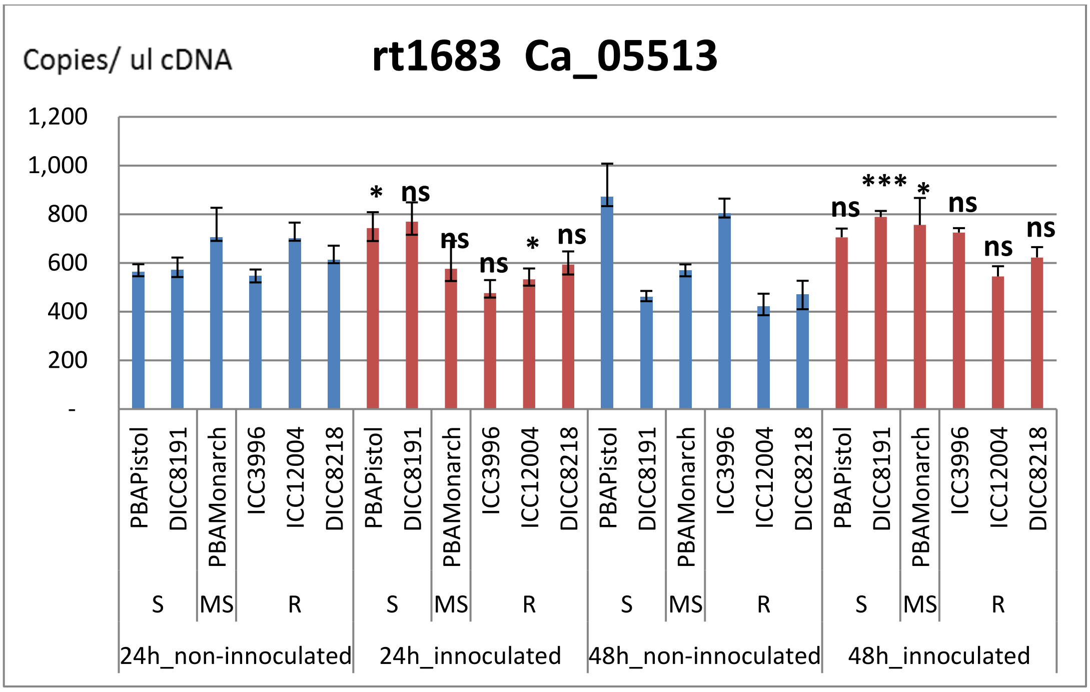

Supplementary Figure 7 **Transcriptional analysis (qPCR) of unknown gene (Ca_05513) in AB4.1 region with 6 chickpea lines at two time points.** Blue: non-inoculated; red: inoculated with *A rabiei*. S: Susceptible; MS: Medium Susceptible; R: Resistant. Significant difference between inoculated and non-inoculated lines are shown as *** (p-value<0.001), * (p-value<0.05). ns: non-significant

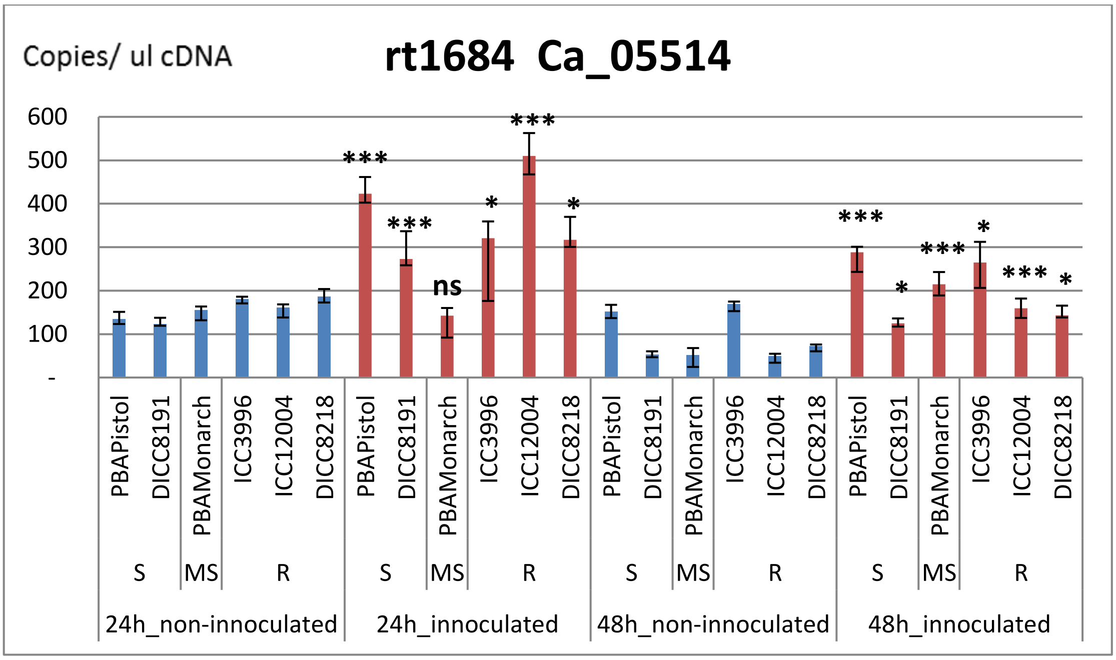

Supplementary Figure 8 **Transcriptional analysis (qPCR) of unknown gene (Ca_05514) in AB4.1 region with 6 chickpea lines at two time points.** Blue: non-inoculated; red: inoculated with *A rabiei*. S: Susceptible; MS: Medium Susceptible; R: Resistant. Significant difference between inoculated and non-inoculated lines are shown as *** (p-value<0.001), * (p-value<0.05). ns: non-significant

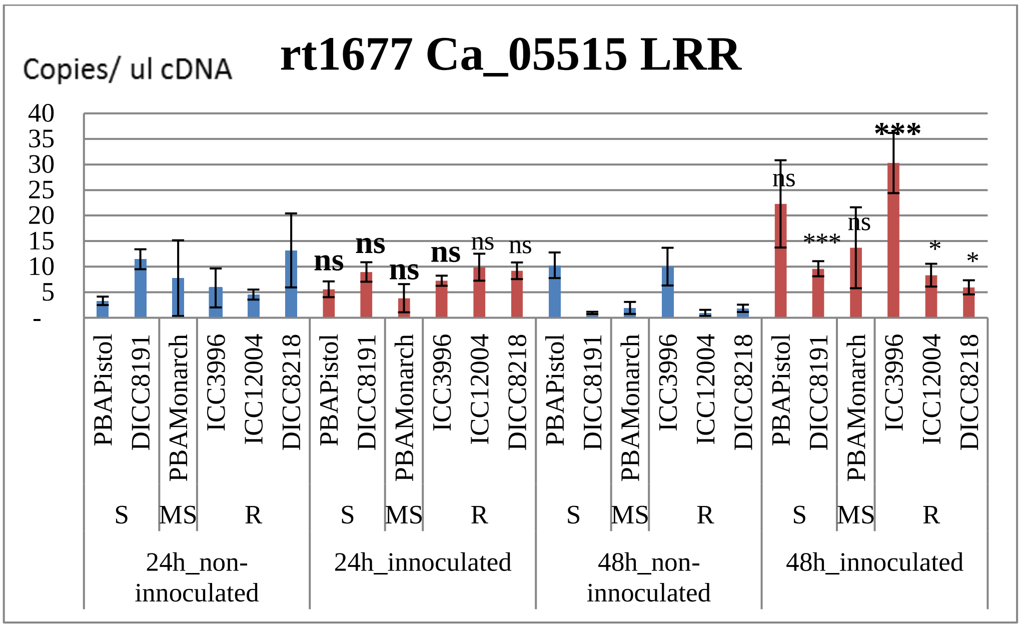

Supplementary Figure 9 **Transcriptional analysis (qPCR) of predicted gene LRR receptor-like kinase (Ca_05515) in AB4.1 region with 6 chickpea lines at two time points.** Blue: non-inoculated; red: inoculated with *A rabiei*. S: Susceptible; MS: Medium Susceptible; R: Resistant. Significant difference between inoculated and non-inoculated lines are shown as *** (p-value<0.001), * (p-value<0.05). ns: non-significant

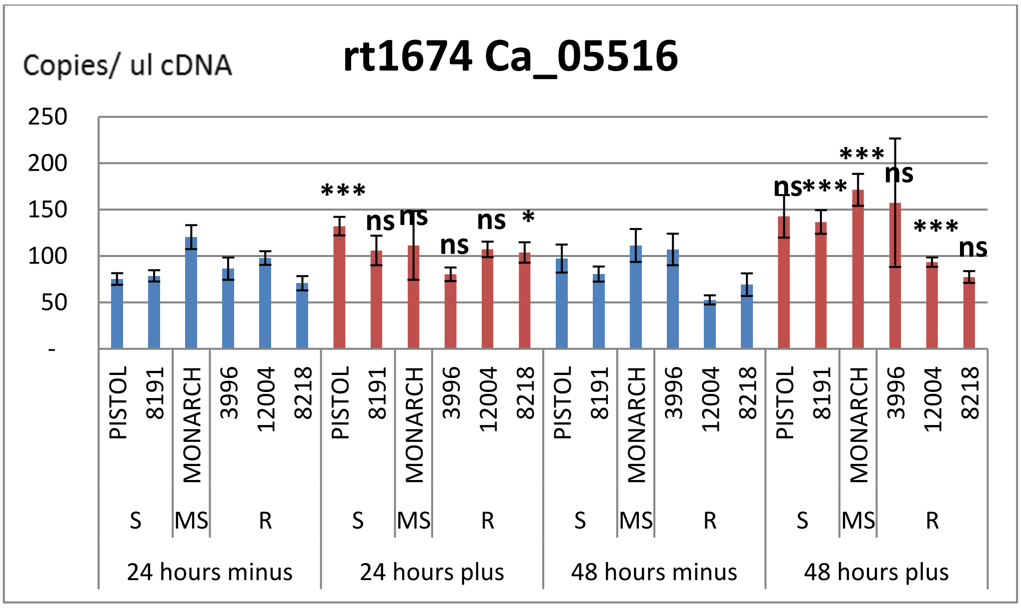

Supplementary Figure 10 **Transcriptional analysis (qPCR) of predicted gene cysteine-rich receptor-like kinase (Ca_05516) in AB4.1 region with 6 chickpea lines at two time points.** Blue: non-inoculated; red: inoculated with *A rabiei*. S: Susceptible; MS: Medium Susceptible; R: Resistant. Significant difference between inoculated and non-inoculated lines are shown as *** (p-value<0.001), * (p-value<0.05). ns: non-significant

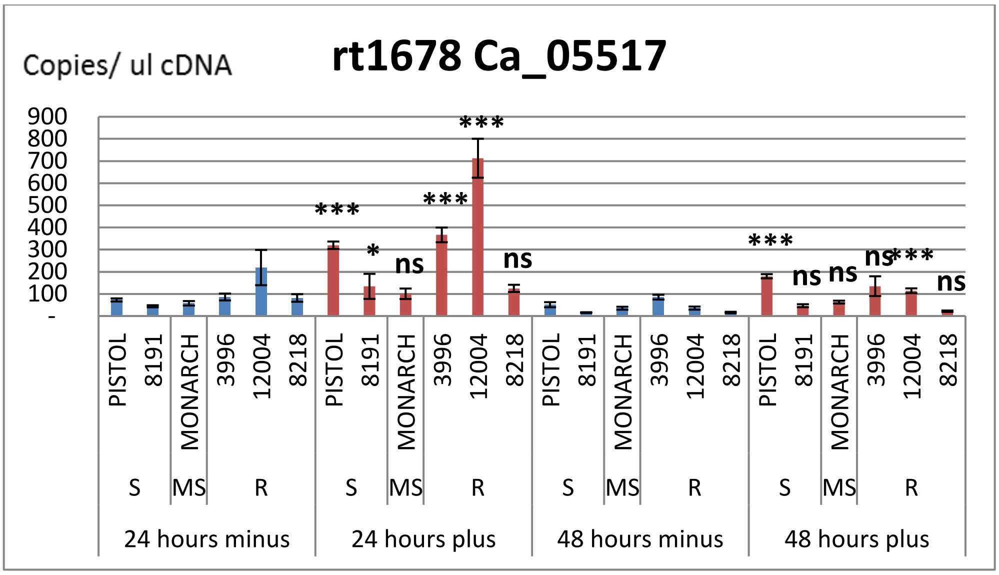

Supplementary Figure 11 **Transcriptional analysis (qPCR) of predicted gene receptor-like kinase (Ca_05517) in AB4.1 region with 6 chickpea lines at two time points.** Blue: non-inoculated; red: inoculated with *A rabiei*. S: Susceptible; MS: Medium Susceptible; R: Resistant. Significant difference between inoculated and non-inoculated lines are shown as *** (p-value<0.001), * (p-value<0.05). ns: non-significant

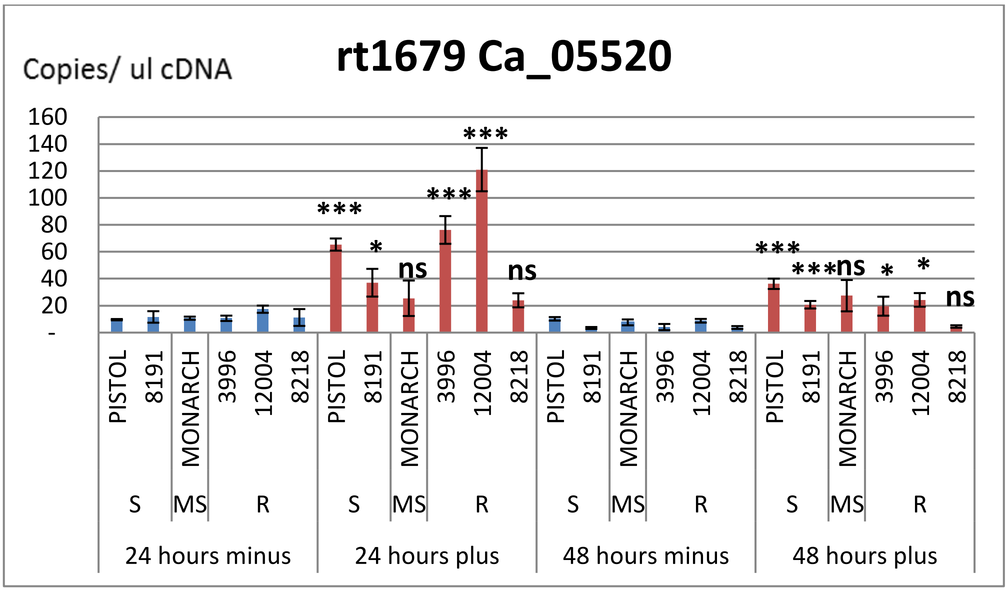

Supplementary Figure 12 **Transcriptional analysis (qPCR) of predicted gene wall associated receptor-like kinase (Ca_05520) in AB4.1 region with 6 chickpea lines at two time points.** Blue: non-inoculated; red: inoculated with *A rabiei*. S: Susceptible; MS: Medium Susceptible; R: Resistant. Significant difference between inoculated and non-inoculated lines are shown as *** (p-value<0.001), * (p-value<0.05). ns: non-significant

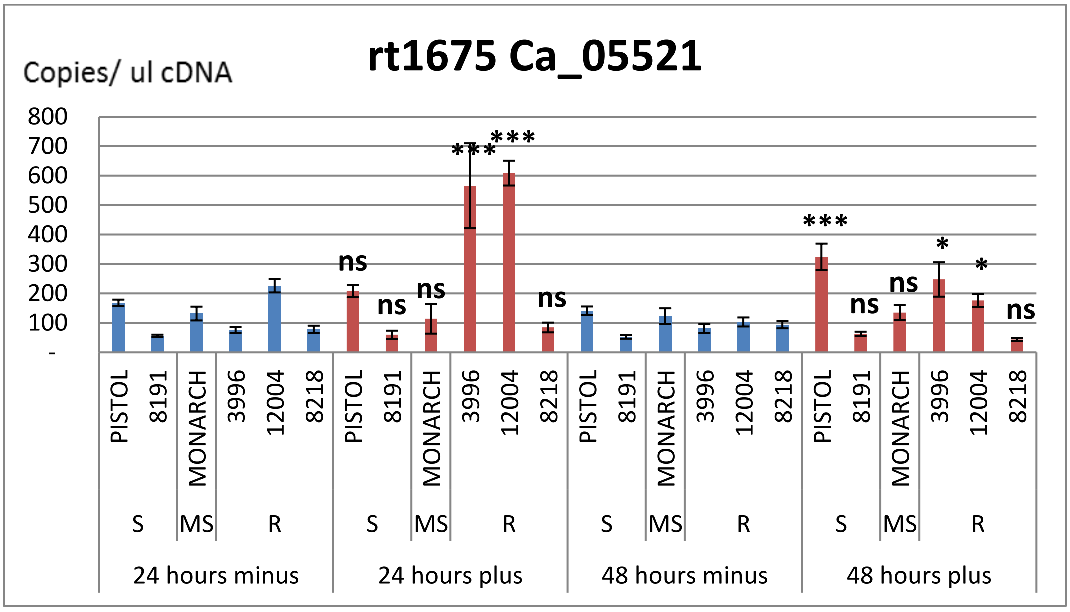

Supplementary Figure 13 **Transcriptional analysis (qPCR) of predicted gene serine/threonine receptor-like kinase (Ca_05521) in AB4.1 region with 6 chickpea lines at two time points.** Blue: non-inoculated; red: inoculated with *A rabiei*. S: Susceptible; MS: Medium Susceptible; R: Resistant. Significant difference between inoculated and non-inoculated lines are shown as *** (p-value<0.001), * (p-value<0.05). ns: non-significant

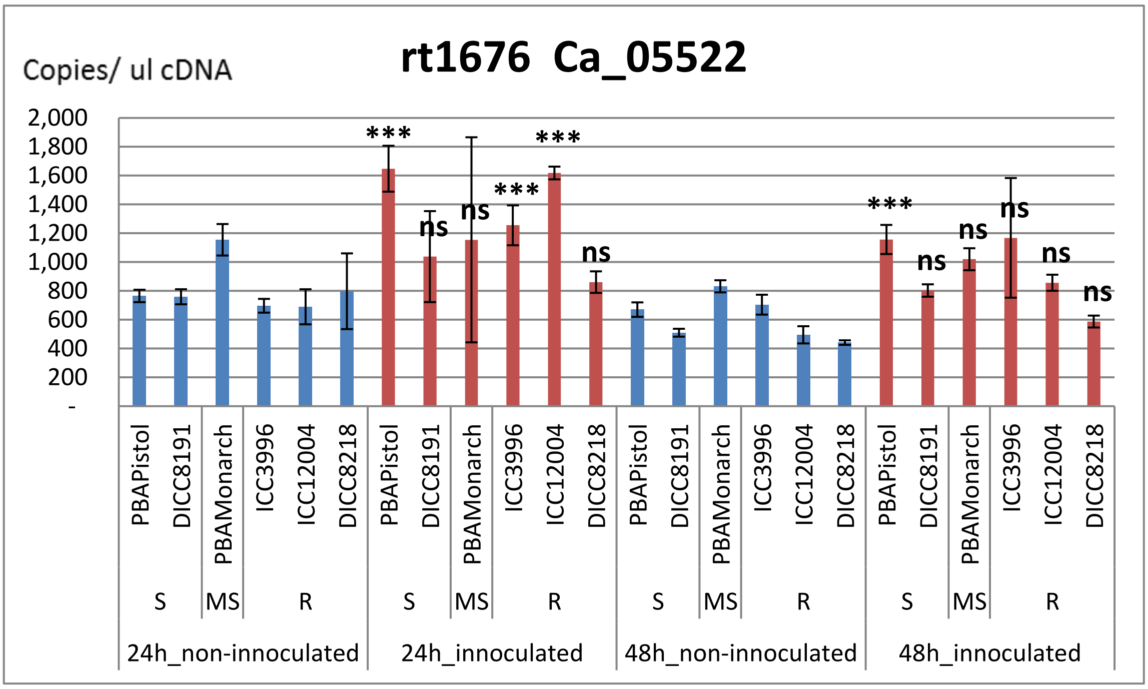

Supplementary Figure 14 **Transcriptional analysis (qPCR) of predicted gene serine/threonine receptor-like kinase (Ca_05522) in AB4.1 region with 6 chickpea lines at two time points.** Blue: non-inoculated; red: inoculated with *A rabiei*. S: Susceptible; MS: Medium Susceptible; R: Resistant. Significant difference between inoculated and non-inoculated lines are shown as *** (p-value<0.001), * (p-value<0.05). ns: non-significant

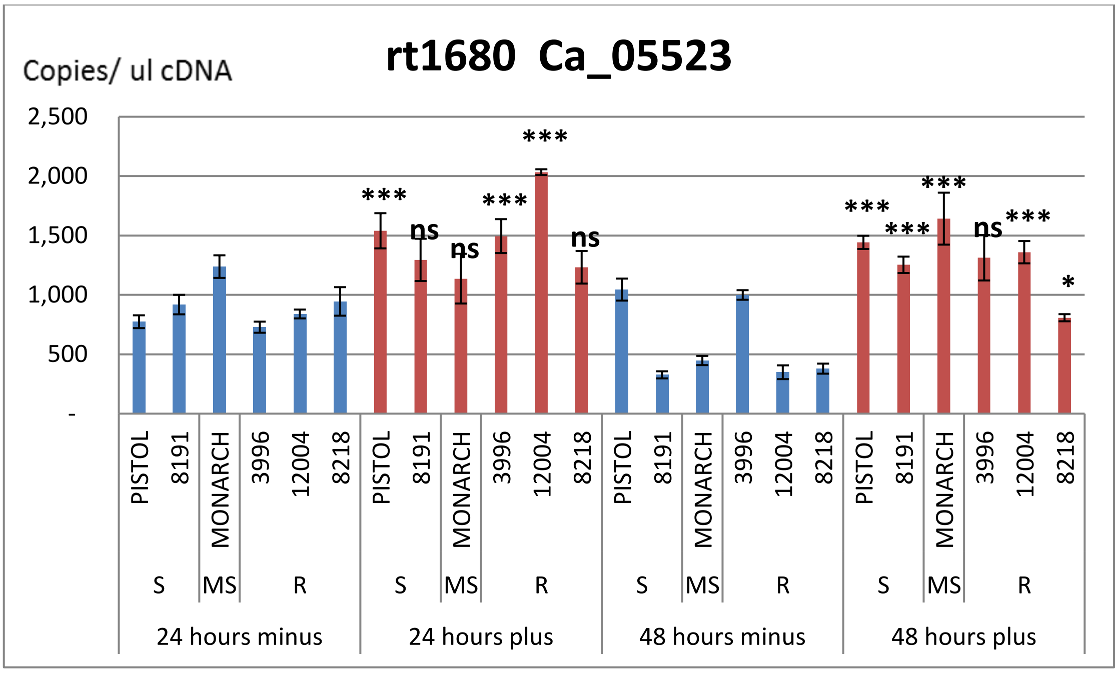

Supplementary Figure 15 **Transcriptional analysis (qPCR) of predicted gene serine/threonine receptor-like kinase (Ca_05523) in AB4.1 region with 6 chickpea lines at two time points.** Blue: non-inoculated; red: inoculated with *A rabiei*. S: Susceptible; MS: Medium Susceptible; R: Resistant. Significant difference between inoculated and non-inoculated lines are shown as *** (p-value<0.001), * (p-value<0.05). ns: non-significant
